# HA1077 displays synergistic activity with daclatasvir against hepatitis C virus and suppresses the emergence of NS5A resistance-associated substitutions in mice

**DOI:** 10.1038/s41598-018-30460-3

**Published:** 2018-08-20

**Authors:** Seung-Hoon Lee, Jae-Su Moon, Bo-Yeong Pak, Geon-Woo Kim, Wooseong Lee, Hee Cho, SangKyu Kim, Seong-Jun Kim, Jong-Won Oh

**Affiliations:** 1 Department of Biotechnology, Yonsei University, Seoul, 03722 Korea; 2 Department of Systems Immunology, Gangwon National University, Gangwon-do, 24341 Korea; 3 Center for Convergent Research of Emerging Virus Infection, Korea Research Institute of Chemical Technology, Daejeon, 34114 Korea

## Abstract

The kinase C-related kinase 2 (PRK2), which phosphorylates hepatitis C virus (HCV) RNA polymerase, is a proviral factor enhancing HCV replication. Here, we report on the *in vivo* anti-HCV efficacy of HA1077, which inhibits viral genome replication by targeting PRK2 and displays viral entry inhibitory activity by targeting Rho-associated kinase. HA1077 showed synergistic antiviral activity selectively with nonstructural protein 5 A (NS5A) inhibitors including daclatasvir (DCV). HA1077 oral administration substantially reduced serum viral loads in mice bearing HCV genotype 2a-replicating Huh7 xenografts. When administered with DCV, HA1077 potentiated the antiviral efficacy of DCV and suppressed the generation of DCV resistance-associated variants (RAVs). By deep-sequencing analysis, we uncovered an unprecedented DCV-induced polymorphism at the poly-proline motif (PxxPxxP) of NS5A. Coadministration of HA1077 reduced such a polymorphism. Overall, our results demonstrate the potential therapeutic benefit of combination therapy with HA1077 plus DCV for HCV patients carrying emerging or pre-existing RAVs toward NS5A inhibitors.

## Introduction

Hepatitis C virus (HCV) is a major cause of chronic liver diseases and hepatocellular carcinoma. Currently, it is estimated that more than 185 million people worldwide are suffering from HCV-related diseases^1^. After the clinical application of the first-generation NS3/4 A protease inhibitors telaprevir and boceprevir, during the last decade a number of direct acting antiviral agents (DAAs) targeting viral nonstructural (NS) proteins [e.g., NS3/4 A protease, NS5A, and NS5B RNA-dependent RNA polymerase (RdRp)] have been approved to treat HCV infection and they have been displaying a high sustained virological response (SVR) rate of up to ~90%^2–6^.

Despite their potent antiviral efficacy, most DAAs, particularly when used as a monotherapy, have a lower genetic barrier to resistance^7^, because HCV RNA polymerase ensures the survival of the virus due to its low fidelity, which leads to high genetic variation in the HCV genome^8^. Furthermore, preexisting DAA resistance-associated variants (RAVs), even in naïve patients, and those selected during DAA combination therapies have been observed in clinical studies^9^. Therefore, emergence of DAA RAVs is yet a major drawback of DAAs. To overcome this, combination therapies using DAAs become now representative approaches for HCV treatment^10–15^. Nevertheless, new combination therapies still need to be developed for patients with RAVs who do not respond to current DAAs and for those with difficult-to-treat HCV genotypes such that alternative or advanced treatment options can be provided for better management of HCV infection^4^.

Host-targeting antiviral agents (HTAs) are potentially pan-HCV genotypic antivirals that have a higher genetic barrier to resistance than DAAs do because they target proviral cellular factors. Thus, HTAs are attractive alternative strategies to overcome the shortcomings or complement the drawbacks of current DAAs. There are two main targets of HTAs: the factors enhancing innate immune responses and the proviral cellular factors necessary for HCV life cycle^16^. Despite of a number of HTAs that have been investigated as treatment options for patients carrying RAVs against DAAs, no agents have been approved for treatment so far^17,18^. We previously demonstrated that protein kinase C-related kinase 2 (PRK2; also known as PKN2) is responsible for the phosphorylation of HCV RdRp^19,20^. Inhibition of PRK2 and its depletion by RNAi reduced HCV loads *in vitro* and in a mouse model of HCV replication, respectively, proving its proviral role^21,22^.

HA1077 (also known as fasudil), which inhibits both PRK2 and Rho-associated kinase (ROCK) with high selectivity compared numerous other kinases^23^, has been used to treat cerebral vasospasm via its ROCK-inhibiting activity^24^. It also delayed cerebral ischemic symptoms and prevented myocardial ischemia in patients with vasospastic angina by targeting ROCK activity^25,26^. In addition, HA1077 displayed tumor metastasis inhibitory activity in human as well as in rat tumor models without noticeable adverse effects^27^. In this study, we set out a strategy to reduce the frequency of DAA resistance mutant emergence by combining DAAs with HA1077. We investigated if there are any synergistic effects by such combinations and analyzed whether HA1077 can suppress the emergence of RAVs against the most potent anti-HCV drug targeting NS5A, daclatasvir (DCV; also known as BMS-790052 and Daklinza)^6^ in a mouse model of HCV replication.

## Results

### HA1077 interferes with HCV entry via its ROCK-inhibitory activity

**Figure 1:**
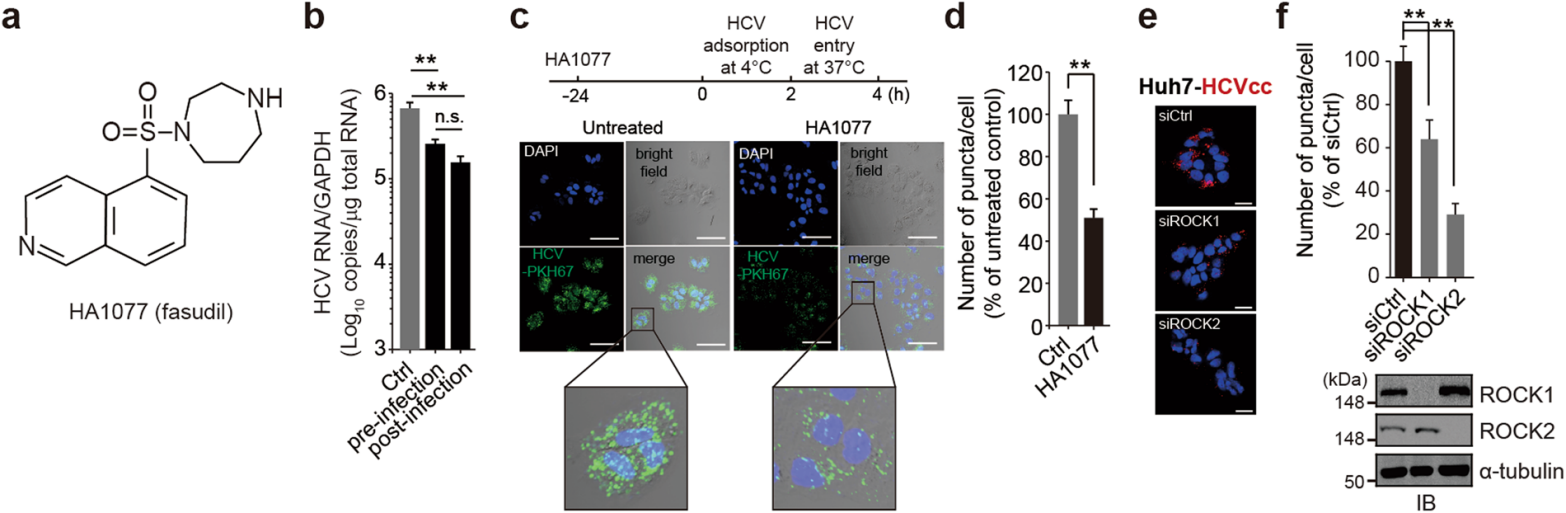
Inhibition of HCV entry by HA1077. (**a**) Molecular structure of HA1077. (**b**) Huh7 cell pre-treated with HA1077 (20 μM) for 24 h were infected with HCV or HCV-infected cells were treated with HA1077. Intracellular HCV RNA loads were determined by RT-qPCR 2 days after viral infection. (**c**,**d**) Entry of PKH67 dye-labeled HCV was monitored in HA1077-pretreated Huh7 cells by fluorescent microscopic analysis (**c**) and quantification of fluorescence intensity (**d**). Images were representative of at least three independent experiments and were taken 6 h post-infection. (**e**,**f**) Effect of ROCK1 or ROCK2 depletion on HCV entry was analyzed as in (**c**,**d**) 2 days after treatment with the indicated siRNAs (50 nM). The immunoblots (IB) are representative of two independent experiments. In all panels, data are mean ± s.d. **P < 0.01 by unpaired two-tailed Student’s *t*-test. n.s., not significant. Scale bar, 50 μm.

Recent genome-wide siRNA screening studies identified ROCK2 as a proviral factor with a potential role in HCV entry^28^. Since HA1077 [1-(5-isoquinoline-sulfonyl)-homopiperazine] (Fig. 1a) was originally discovered as a ROCK inhibitor although it is equally active against PRK2^23,24^, we were interested in testing the impact of HA1077 on HCV entry. As shown Fig. 1b, pre-treatment with HA1077 significantly reduced HCV RNA titers in HCV-infected Huh7 cells, although the degree of inhibition was slightly lower, albeit not statistically significant, than that achieved by post-infection HA1077 treatment. Further virus entry experiments using PKH67 dye-labeled HCV revealed that HA1077 could indeed impede HCV entry as evidenced by the ~50% decrease in HCV-positive staining in HA1077-pretreated cells (Fig. 1c,d).

We previously observed that RNAi-mediated depletion of ROCK1 or ROCK2 had no effect on viral replication in Huh7 cells harboring an HCV subgenomic replicon^21^. However, silencing the expression of ROCK1 or ROCK2 prior to HCV infection interfered with HCV entry (Fig. 1e,f), verifying the important function of these kinases in the HCV entry process and demonstrating that anti-HCV activity of HA1077 is in part due to the inhibition of ROCK-mediated HCV entry. HA1077 also displayed anti-HCV activity by inhibiting PRK2-mediated HCV RNA polymerase phosphorylation^22^. Thus, the anti-HCV activity of HA1077 appears to be mediated through its dual inhibitory activity against PRK2 and ROCK.

### Therapeutic efficacy of HA1077 in a mouse model of HCV replication

**Figure 2:**
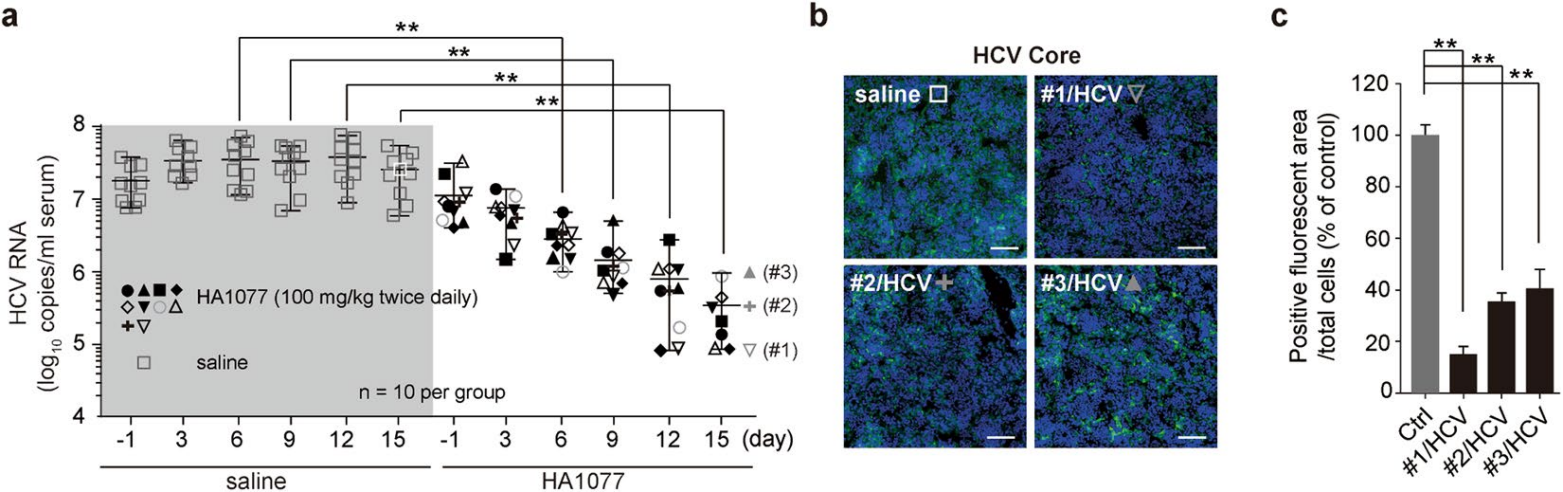
Antiviral efficacy of HA1077 in an orthotopic xenograft mouse model of HCV replication. (**a**) Serum HCV RNA titer in NOD-SCID mice carrying HCV-infected Huh7 cells implanted in the liver. The mice were given saline or HA1077 (100 mg/kg body weight) perorally twice a day for 15 days. (**b**,**c**) Immunostaining of HCV core protein (Core) in the xenografts retrieved at day 15 from the three randomly picked mice receiving HA1077 [#1, 2, and 3 marked in (**a**)] or the control mouse receiving saline (**b**). Quantification of Core-positive cells using the ImageJ program (**c**). Ctrl, sample from saline-receiving mice. Scale bars, 100 μm. In all panels, data are mean ± s.d. **P < 0.01 by unpaired two-tailed Student’s *t*-test.

We tested the antiviral activity of HA1077 in immunocompromised non-obese diabetic/severe combined immunodeficiency (NOD-SCID) mice bearing HCV-infected Huh7 cells, which were implanted orthotopically into the mouse liver^21^. The mice were given HA1077 perorally every 12 hours based on its *in vitro* PRK2 inhibitory activity lasting 8–12 h, as reflected by pPRK2 level reduction assessed by immunoblotting analysis (Supplementary Fig. 1). Oral administration of HA1077 (100 mg/kg body weight) twice daily with a 12-h interval significantly reduced serum HCV RNA loads (94% reduction compared to saline-treated group, **P < 0.01) at day 6 (Fig. 2a). HA1077 displayed various degrees of antiviral activity in all of the treated mice (*n* = 10) and resulted in a mean reduction of 1.9 log_10_ serum HCV titer with a maximum 2.9 log_10_ viral titer reduction at day 15. Consequently, we could observe a significant reduction in HCV core protein levels at different depths (upper, middle, and lower portions) of the retrieved xenografts of HCV-infected Huh7 (Fig. 2b,c; overall 60–85% reduction in three randomly selected mice, **P < 0.01) (Fig. 2b,c).

### HA1077 synergistically inhibits HCV with NS5A inhibitors

**Figure 3:**
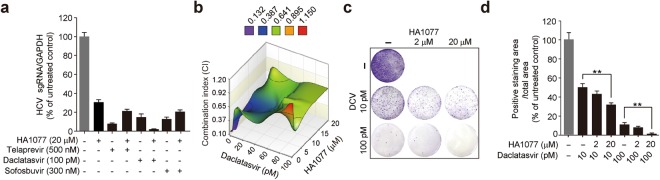
Antiviral efficacy of combination of HA1077 and HCV direct-acting antiviral agents (DAAs). (**a**) Huh7-derived R-1 cells harboring a GT1b HCV subgenomic replicon was treated with HA1077 (20 μM) and indicated HCV DAAs (EC_90_ of each compound). Two days after drug treatment, intracellular HCV load was determined by RT-qPCR. Data are mean ± s.d. from of three independent experiments, each involving triplicates. (**b**) Synergy effect of combination of daclatasvir (DCV) and HA1077 was assessed by calculating the combination index (CI) values for a representative experiment. CI < 1, synergism; CI = 1, additive effect; and CI > 1, antagonism. (**c**,**d**) Suppression of DAA-resistant mutant generation by combining HA1077 with DCV. R-1 cells were cultivated in the presence of G418 (0.5 mg/ml) and the indicated combinations of HA1077 and DCV for 5 weeks to select for DCV resistant mutants (**c**). Quantification of drug-resistant colonies using the ImageJ program (**d**). **P < 0.01 by unpaired two-tailed Student’s *t*-test.

Since HCV clearance was not achieved by HA1077 alone in the 2-weeks administration schedule, we assessed whether its antiviral efficacy could be potentiated in synergy with DAAs. Initially, we chose the NS3/4 A protease inhibitor telaprevir, the NS5A inhibitor DCV, and the NS5B inhibitor sofosbuvir as combinatory partners of HA1077. Among these DAAs tested at their EC_90_ values with 20 μM HA1077, only DCV synergistically reduced HCV subgenomic replicon levels (Fig. 3a,b). By contrast, telaprevir and two additional protease inhibitors (simeprevir and asunaprevir: Supplementary Fig. 2) did not show synergy when used in various combinations with HA1077.

In addition to DCV, two other potent NS5A inhibitors, ledipasvir and ombitasvir, showed such a synergy effect (Supplementary Fig. 3). These NS5A inhibitors reduced HCV RNA titer effectively in synergy with HA1077 at various tested doses [combination index (CI) value of <1 at all tested combinations]. In contrast, none of the NS5B inhibitors tested (i.e., sofosbuvir, dasabuvir, and mericitabine) synergistically potentiated the antiviral activity of HA1077 (Fig. 3a and Supplementary Fig. 4). Importantly, the combination of DCV and HA1077 effectively prevented the generation of DCV-resistant variants as revealed by the colony formation assay using an HCV genotype (GT) 1b subgenomic replicon; combination of 100 pM DCV and 20 μM HA1077 reduced the resistant variant generation frequency from 11% (by DCV alone) to 1.5% (Fig. 3c,d).

### *In vivo* efficacy of the HA1077 and DCV combination against HCV

**Figure 4:**
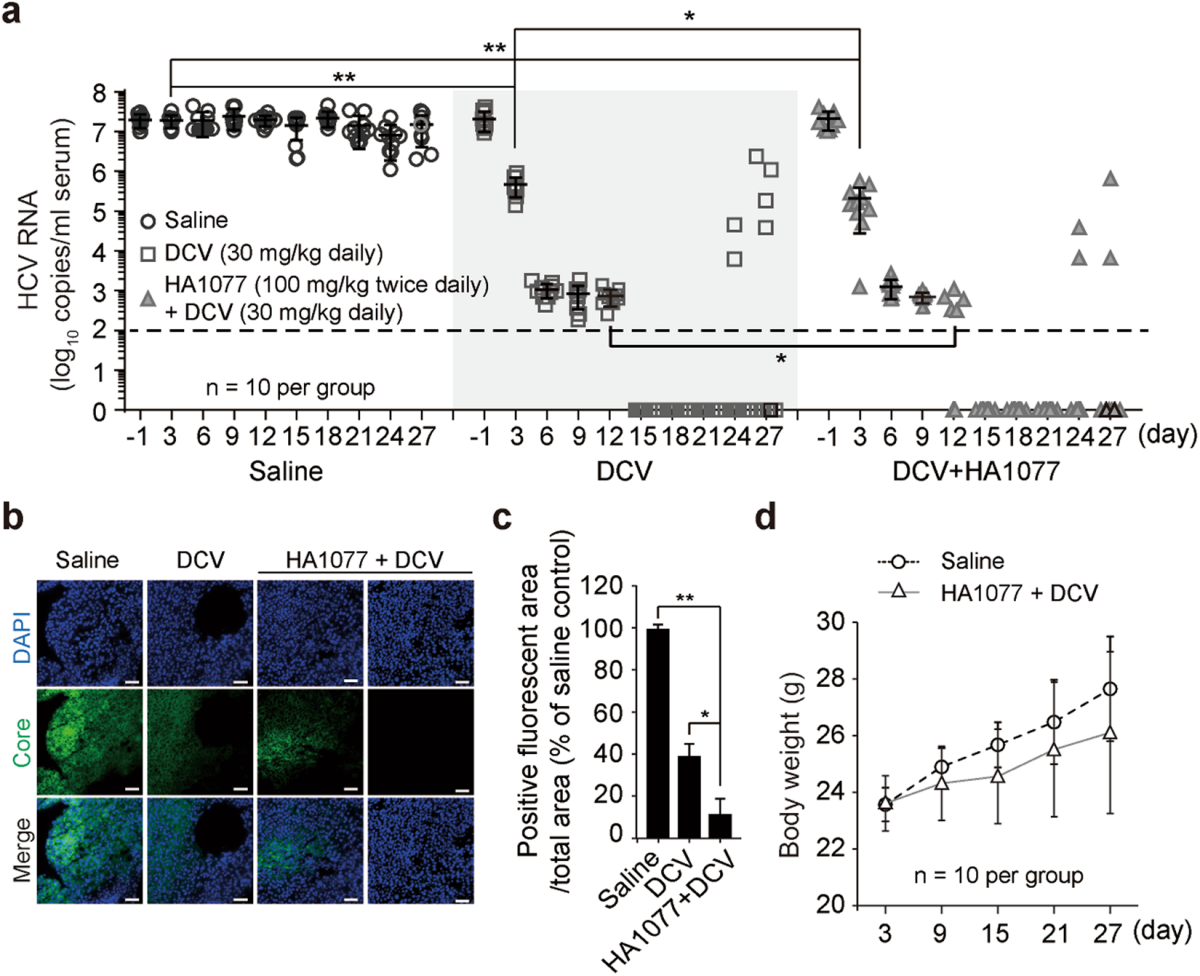
HA1077 coadministered with DCV suppresses the emergence of DCV-resistant mutants in an orthotopic xenograft mouse model of HCV replication. (**a**) Saline or DCV (30 mg/kg body weight; once a day) with or without HA1077 (100 mg/kg body weight; twice with a 12-h interval per day) was given to mice (n = 10 per group) for 27 days. Serum HCV RNA titer was monitored every 3 days by RT-qPCR. The horizontal dotted line indicates the detection limit (2 log_10_/ml) of the TaqMan real-time RT-qPCR assay. (**b**,**c**) Immunostaining for HCV core protein (Core)-positive cells in the xenografts retrieved at day 27 (**b**). Shown are the representative images at 3 different depths of the harvested xenografts of HCV-infected Huh7 (5×5×5 mm). Quantification of Core-positive cells (*n* = 3) using the ImageJ program (**c**). (**d**) Body weight changes in mice treated as described in (**a**). *P < 0.05; **P < 0.01; by unpaired two-tailed Student’s *t*-test. Scale bar, 50 μm.

DCV displays its anti-HCV activity by multiple mechanisms including disruption of replication factory formation and prevention of viral genome transfer to assembly sites^16,29–31^. Currently, it is the most potent antiviral agent used for HCV GT 1a/1b patients^6^. We tested the impact of HA1077 plus DCV combination therapy in a NOD-SCID mouse model of HCV replication. As expected, DCV administered alone perorally (once a day at 30 mg/kg body weight) reduced serum HCV RNA titers very rapidly by ~1.5 log_10_ at day 3 (Fig. 4a), proving its superior efficacy. When HA1077 (twice daily at 100 mg/kg body weight) was administered with DCV, the viral titer decreased further by 74% compared with the titer achieved by DCV alone. Notably, the combination of DCV with HA1077 potentiated the antiviral efficacy of HA1077 (a mean reduction of 1.9 log_10_ in HCV titer by HA1077 vs. viral clearance by the combination therapy on day 15; compare the results in Figs 2a and 4a).

The rapid and steep decline in HCV RNA levels was followed by a slower second phase decline until day 12 to result in clearance of HCV (below the detection limit of the TaqMan RT-qPCR assay) in five mice receiving both HA1077 and DCV. In all mice in the DCV alone-treated group, HCV titers decreased below the detection limit at day 15. This suppression was maintained until day 21 in both DCV- and DCV plus HA1077-treated groups. Both DCV and DCV plus HA1077 oral administrations over 27 days were generally tolerated with noticeable adverse effects. However, HCV rebound was observed in two mice in both groups at day 24 and additional HCV rebound was further observed in two mice only in the DCV-receiving group at day 27. HCV rebound could not be monitored further because experiments had to be terminated due to the increase of the tumor volume above the limit set by the guidelines of animal care ethics. Immunostaining analysis of the xenografts retrieved at day 27 showed a significant reduction of HCV core protein-positive cell levels (61% and 89.1% reduction in DCV-treated group and DCV plus HA1077-treated group compared with the saline-treated control group, respectively) (Fig. 4b,c). The mice that received DCV plus HA1077 had a slightly reduced body weight compared with mice in the saline-administered group, although statistically insignificant (Fig. 4d).

### Deep sequencing analysis for DCV resistance-associated substitutions on NS5A

In order to analyze the profile of DCV resistance-associated substitutions (RASs) on NS5A, we terminated the experiment on day 27. We selected three mice from the saline-treated group (with serum HCV RNA titer of 7.1 × 10^7^, 7.3 × 10^7^, and 6.3 × 10^7^/ml at day 27), four mice from the DCV-treated group (with serum HCV RNA titer of 2.1 × 10^6^, 1.0 × 10^6^, 2.0 × 10^5^, and 3.5 × 10^4^/ml at day 27), and two mice from the DCV plus HA1077-treated group (with serum HCV RNA titer of 6.8 × 10^3^ and 7.4 × 10^5^/ml at day 27). The six mice in the last two groups had serum HCV RNA titers remained over 5 × 10^3^/ml from day 24. In addition to these three groups, five mice from the DCV- or DCV plus HA1077-treated group (with serum viral loads below the detection limit of the RT-qPCR assay at day 27) were chosen for deep-sequencing analysis of DCV RAVs. We also included five mice that received HA1077 for 15 days (100 mg/kg body weight twice daily; see Fig. 2a) alone to assess its impact on NS5A RASs.

**Figure 5:**
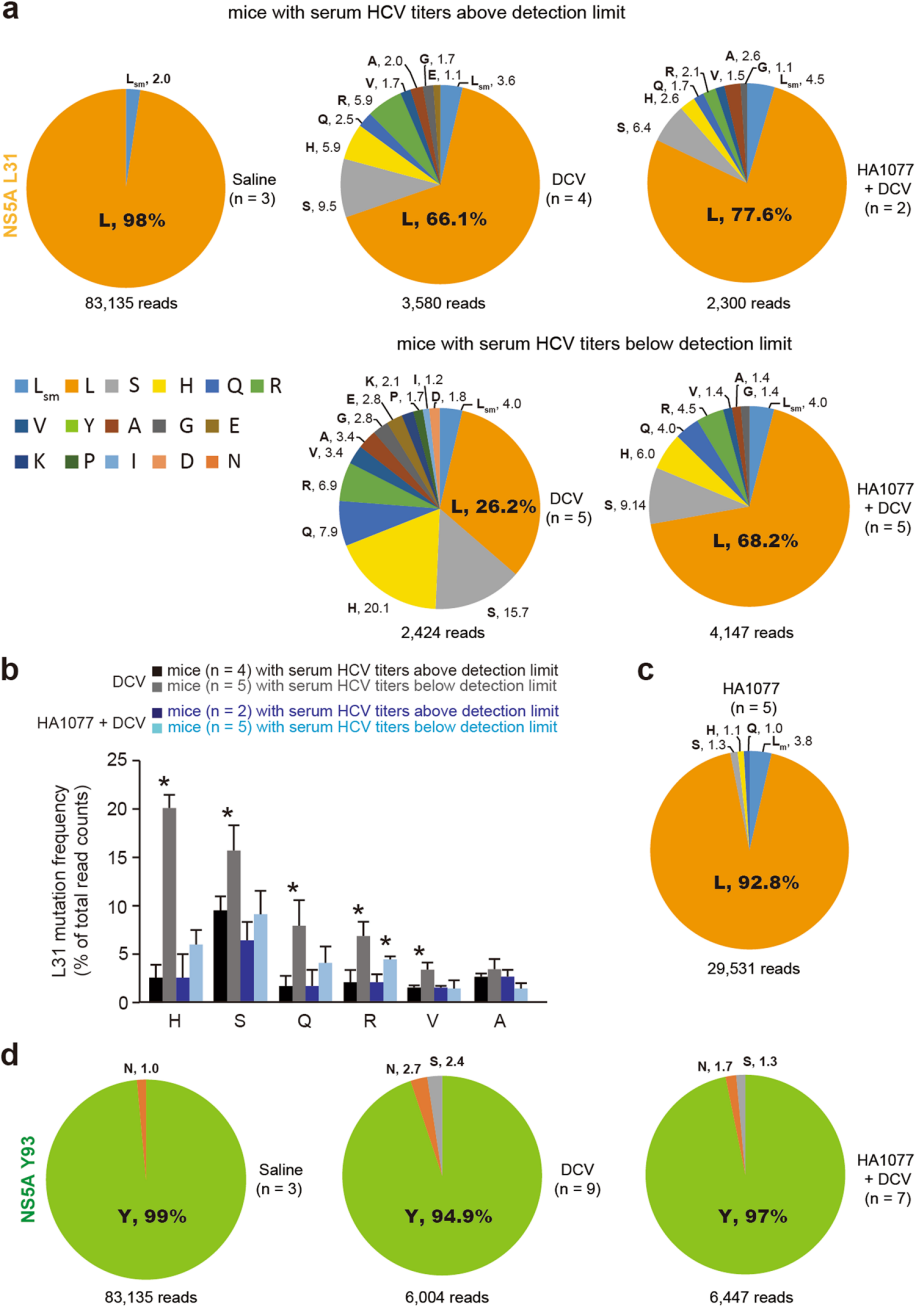
NS5A L31 and Y93 polymorphism associated with DCV resistance. (**a**) DCV-induced polymorphism on NS5A L31 in the HCV GT2a strain JFH1. Illumina MiSEQ sequencing analysis was carried out using total RNA from HCV-infected Huh7 xenografts, which were recovered from the indicated mice receiving saline, DCV, or HA1077 plus DCV for 27 days. sm, silent mutation. (**b**) Mutation frequency on various L31 substitutions in mice with HCV rebound or with undetectable serum HCV titers after administration of DCV or DCV plus HA1077 for 27 days. ND, not detected. Asterisks indicate the residues that show a significant increase or decrease in mutation frequency. *P < 0.05 by unpaired two-tailed Student’s *t*-test. (**c**) L31 polymorphism in mice receiving HA1077 for 15 days. (**d**) Y93 polymorphism was analyzed as in (**a**).

In an attempt to explore the NS5A polymorphism even in mice with undetectable serum HCV titers, we used the HCV-infected Huh7 xenografts from which we could isolate enough total RNA for PCR amplification of the cDNA covering the L31 and Y93 residues, the two well-characterized DCV RASs on NS5A. We were able to obtain amplicons for sequencing analysis when we used total RNA (5 μg) extracted from the xenografts retrieved from the mice, which received DCV alone or DCV plus HA1077 and had HCV RNA titers below detection limit when assays were performed using total RNA from 200 μl sera from these mice. By sequence analysis of the resulting amplicons using an Illumina MiSEQ sequencer, we found no amino acid substitutions on the L31 residue in saline-treated group, although silent mutations were found at a 2.0% frequency (Fig. 5a). The silent mutations on L31 were also found in drug-treated groups with a 3.6–4.5% frequency, suggesting that L31 might be a hypermutable site even in the absence of any selection pressures. Unexpectedly, L31M, which is the most frequent substitution found in HCV GT1 DCV RASs, was not detected in this HCV GT2a (JFH1) strain. By contrast, the substitution frequency at the L31 residues was dramatically increased by 33.9% in mice with HCV rebound in the DCV-treated group. Such an expansion of amino acids variations on the L31 residue was not previously observed when similar analysis was conducted on the sera from HCV GT1 patients^13,32,33^.

A strikingly surprising finding was that L31 substitution frequency increased further in mice with undetectable serum HCV titers compared with that in mice showing HCV rebound (33.9% with 8 different substitutions vs. 73.8% with 12 different substitutions). Of note, we noticed that when HA1077 was administered together with DCV, these increased substitution frequencies were substantially reduced from 73.8% to 31.8% in mice with undetectable serum HCV titers (**P < 0.05 by two-tailed, nonparametric Mann-Whitney test) and from 33.9% to 22.4% in mice with HCV rebound (*n* = 2; HCV titers over 5 × 10^3^/ml from day 24). For the top five most frequent substitutions at the L31 residue, the mutation frequency was significantly increased in the mice (*n* = 5) showing undetectable serum HCV titers following DCV treatment (Fig. 5b). Such an expansion of polymorphism on L31 induced by DCV was dramatically suppressed by HA1077, suggesting its role in suppressing DCV RAV generation.

Unexpectedly, similar analysis of the mice (*n* = 5) receiving HA1077 alone showed the emergence of L31 variants (L31H/Q/S with a 3.4% frequency in total) (Fig. 5c). Interestingly, those three substitutions were the major mutations that were also found in mice with undetectable serum HCV titers. Furthermore, silent mutation frequency also increased from 2% in saline-treated mouse group to 3.8% in HA1077-receiving mouse group. These results suggested that L31 residue might be intrinsically hypervariable and raised the possibility that HA1077 might target NS5A. Despite its high polymorphism, L31M substitution, which was previously found in GT2a, GT6a, GT1a/b^32–36^, was not detected.

For GT1b, Y93N/H substitution is another major resistance-associated mutation toward DCV^33,36^. In contrast to the high polymorphism observed at the L31 residue, no silent mutations were detected on Y93, and Y93N substitution was detected only at a frequency of 1.0% in the saline-treated control group (Fig. 5d). Nevertheless, Y93N and Y93S substitution frequency increased to 2.7% and 2.4%, respectively in the DCV-treated group, and their mutation frequency was slightly decreased by co-treatment with HA1077.

Collectively, the results from deep-sequencing analysis indicate that the L31 residue of NS5A undergoes rapid mutation under the selection pressure by DCV, and that the emergence of diverse L31 mutants can be suppressed substantially, albeit not completely, by HA1077.

### Hyperpolymorphism of the poly-proline motif (PxxPxxP) in DCV RAVs of HCV GT2a

A predicted docking model for DCV to NS5A suggested binding through Q30 in GT1a and R30 in GT1b^37^. Furthermore, DCV RAVs showed clinically relevant mutations in the vicinity of these binding sites, including M28, L31, and P32 in GT1a (in addition to Q30, H54, and Y93) and L28, L31, P32, and F37 in GT1b (in addition to R30, Q54, P58, and Y93)^13,32,33^. For these reasons and based on our identification of strikingly diverse substitutions on L31, we further explored the polymorphism at the N-terminal NS5A region spanning aa 28–40, downstream of the N-terminal endoplasmic reticulum (ER) membrane anchoring amphipathic α-helix (AH; aa 1–27)^38^. This region includes a conserved poly-proline motif (PxxPxxP) that acts as a linker of the AH at the domain I of NS5A (aa 1–213)^39^. The polymorphism profile of this region are shown in Supplementary Fig. 5.

**Figure 6:**
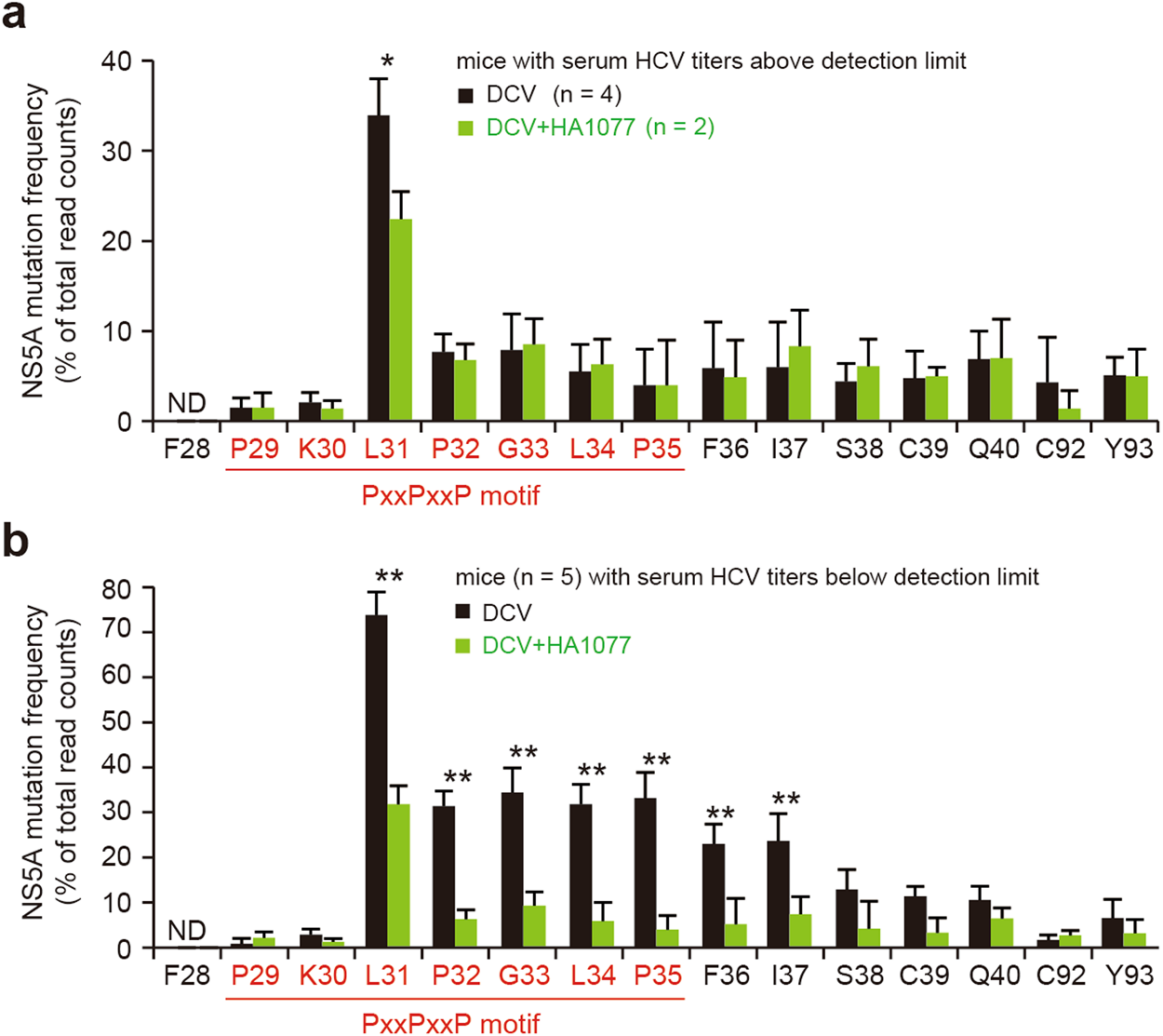
Mutation frequency at the potential DCV-binding sites on NS5A. (**a**,**b**) Frequency of mutations at the residues spanning amino acids 28–40 including the PxxPxxP motif at the downstream of the N-terminal ER membrane-anchoring amphipathic α-helix (AH; aa 1–27), and the two residues C92 and Y93 known to confer DCV resistance in HCV GT2a. Shown are the NS5A polymorphism and DCV RAV emergence-suppressing effect of HA1077 in mice with HCV rebound (**a**) or in mice with undetectable serum HCV titers (**b**) after oral administration of DCV or DCV plus HA1077 for 27 days. *P < 0.05; **P < 0.01; by unpaired two-tailed Student’s *t*-test. ND, not detected.

For GT1a, among various substitutions on Q30 (Q30E/H/K/R) in addition to L31 (L31M/V) and Y93 (Y93C/E/H/N), which were identified as frequent resistance-associated mutations by drug resistant replicon selection experiments *in vitro*, the highest level of resistance was observed with mutants carrying a Q30E (or Y93N) substitution, resulting in several tens of thousands-fold increases of DCV EC_50_^32,33^. In GT2a, this residue was not conserved and the corresponding residue K30 in the JFH1 strain was not substantially mutated (Fig. 6; see the details in Supplementary Fig. 5a).

Whilst lower than the extraordinary high substitution frequency (33.9%) at L31, the residues spanning from P32 to Q40 also displayed ~3.1–7.9% variations in the mice (*n* = 4) with HCV rebound after 27 days of DCV administration (Fig. 6a). In contrast, the L31 upstream residues F28, P29, and K30 displayed no (F28) or very low levels of variation (P29 and K30 with mutation frequency of 1.2% and 2.2%, respectively). Strikingly, L31 polymorphism further increased to 73.8% in the mice (*n* = 5) showing undetectable serum HCV titers following DCV treatment (Fig. 6b). Similar to the results in the mice with HCV rebound, only a marginal variation was observed at the F28, P29, and K30 residues. However, other vicinity residues (P32, G33, L34, P35, F36, and I37) displayed a mutation frequency of >20%, which is much higher than that in the mice with HCV rebound (see also Supplementary Fig. 5b–d). In both mice with HCV rebound and mice with SVR, mutation frequencies at S38, C39, and Q40 as well as C92 and Y93 were below 10% (Fig. 6a,b and Supplementary Fig. 5e,f). In contrast, in the mice that had undetectable serum HCV titers, over 30% mutation rates were detected in the five residues within the PxxPxxP motif, highlighting that the DCV-induced polymorphism was further expanded in a residual, uncleared viral population in the xenografted tissues even when there were undetectable levels of HCV in sera.

Notably, HA1077 co-administered with DCV significantly reduced the L31 mutation frequency in both the two mice with HCV rebound and the five mice, which had undetectable levels of HCV in sera (Fig. 6a,b). In the mice showing undetectable serum HCV titers, the mutation-suppressing effect of HA1077 was even greater (>3-fold reduction) at the P32, G33, L34, P35, F36, and I37 residues than at the L31, thus demonstrating its capability of suppressing the DCV-induced hyperpolymorphism at the linker region of NS5A.

### Replication competence of GT2a DCV RAVs and their responsiveness to DCV and HA1077

**Figure 7:**
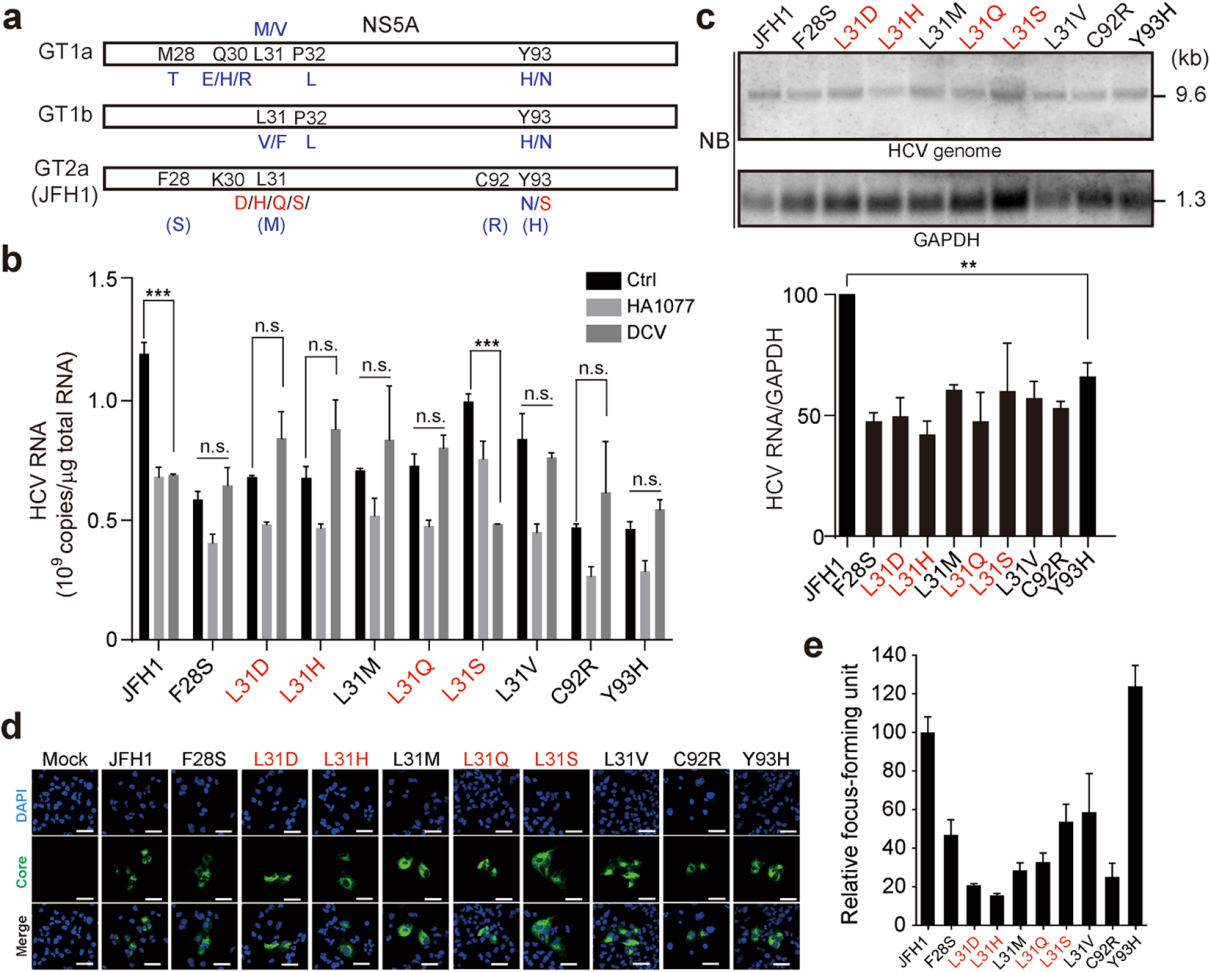
Impact of various substitutions on NS5A L31 and Y93 in HCV replication and responsiveness to DCV and HA1077. (**a**) DCV resistance-associated substitutions in NS5A of HCV GT1a/b and GT2a strains. Shown in black and blue are the amino acid substitutions most frequently identified in the past, and in red the previously uncovered residues identified in this study. The residues shown in parenthesis are the mutations identified previously in GT2a but not in this study with JFH1. (**b**) Susceptibility of JFH1 derivatives with the indicated substitutions to HA1077 and DCV. Antiviral activity of HA1077 (20 μM) and DCV (100 pM) against wild-type JFH1 and its various derivatives was assessed by quantification of HCV RNA titer by RT-qPCR 3 days after transfection of viral RNA transcripts into Huh7 cells. Data are representative of three independent experiments, each involving triplicates. The significance of differences in means between groups was analyzed by one-way analysis of variance (ANOVA) with Bonferroni’s multiple comparisons test. ***P ≤ 0.001; n.s, not significant. (**c**) Replication competence of the transfected HCV transcripts described in (**b**) was assessed by northern blotting. Shown at the top is a representative blot. HCV blots were quantified using a PhosphorImager and normalized to GAPDH expression. Shown at the bottom panel are the relative HCV replication levels (mean ± s.d.) estimated from two independent experiments. **P ≤ 0.01 by unpaired Student’s *t*-test. (**d**,**e**) Infectivity of JFH1 derivatives bearing a DCV resistance-associated NS5A substitution. Huh7 cells were infected with an equal volume of culture supernatants from HCV RNA-transfected cells recovered at 3 days post-transfection, prior to immunostaining of HCV core protein (Core; green) 2 days later. DAPI, nuclear staining (blue) (**d**). Relative percentages of Core-positive cells in HCV-infected Huh7 cells (**e**). The data shown are the means ± s.d. (relative infectivity of each mutant), obtained from three technical replicates. Scale bar, 50 μm.

Deep-sequencing analysis revealed that the repertoire of DCV RAS in HCV GT2 is strikingly different from that in HCV GT1, in which L31M/V and Y93H are major substitutions (Fig. 7a)^34,35,40^. We observed a high L31D/H/Q/S substitution frequency and a very low frequent Y93N/S substitution (at most 2.7%) in the HCV GT2a strain JFH1. Accordingly, we sought to verify whether these major mutations on L31 confer resistance toward DCV. Since F28S, L31M, C92R, and Y93H are the major substitutions identified by resistance selection studies with the JFH1 replicon^35^, we also constructed control JFH1 variants containing those mutations, along with a mutant with an L31V substitution found in GT1a/b. As shown in Fig. 7b, all the tested variants except the one with an L31S substitution became insensitive to DCV, thus confirming the resistance-conferring function of L31/D/H/Q substitutions. These results suggest that some of L31 variants emerged upon DCV treatment may not lead to NS5A phenotype alterations. It still remains to be determined whether HCV containing L31S substitution acquires DCV resistance when linked to other mutations. Unlike DCV, HA1077 limited the replication of these mutants at various degrees.

As revealed by a transient HCV replication assay using *in vitro* transcripts of HCV variants, all of the tested variants described above had reduced replication capability, showing a 34–58% reduction in HCV RNA levels compared to the parental virus JFH1 strain (Fig. 7c). Infectious virus titers of these variants, except the one with an Y93H substitution, were also reduced as demonstrated by the infection experiments with HCVcc harvested three days after viral RNA transfection (Fig. 7d,e). The slightly increased infectious virus titer of the Y93H variant is reminiscent of the previous finding with a chimeric JFH1 replicon carrying an HCV GT1b NS5A(Y93H)^36^. Overall, our results identify the L31D/H/Q as novel DCV RASs in HCV GT2a and suggest that there exist much more diverse repertoires of substitutions on L31 and its vicinity residues than ever expected to allow HCV to survive in the presence of the selection pressure induced by DCV.

## Discussion

Despite the availability of multiple potent DAAs to treat major genotypes of HCV, they have an intrinsic pitfall of rapid emergence of drug-resistance variants, except a small number of nucleoside NS5B inhibitors, due to the high turnover rate of HCV viral RNA genome and error-prone nature of the viral RNA polymerase. Therefore, there is a clear unmet medical need for new therapies that can overcome the shortcomings of DAAs. In this study, we demonstrated that HA1077 monotherapy effectively limits HCV despite high baseline viral load (>10^7^/ml) in an immunocompromised mouse model of HCV replication. HA1077 improved the therapeutic efficacy of DCV and suppressed the emergence of DCV RAVs. Such a synergistic effect was observed exclusively with NS5A inhibitors but not with NS3/4A protease or NS5B RdRp inhibitors. Currently, emergence of DCV RAVs in HCV GT2 has not been studied thoroughly, and available data are from *in vitro* studies using HCV replicon systems^34,35^ and from very limited clinical trials^35,36^. Our deep-sequencing analysis of HCV variants in the immunocompromised NOD-SCID mice, which would enable HCV to propagate more aggressively due to the lack of host immune responses, identified previously uncovered DCV RASs clustered in the PxxPxxP motif in HCV GT2a NS5A. HA1077 suppressed the emergence of such polymorphic NS5A variants.

HCV NS5A, which is a phosphoprotein with RNA-binding activity, is a multifunctional protein essential for viral genome replication and virion assembly^41,42^. DCV inhibits the viral replicase complex formation and interferes with virion assembly possibly by blocking viral genome transfer to an infectious virus assembly site^29–31^. Due to its multifunctional roles at various stages of the HCV life cycle, multiple reasons might explain the NS5A inhibitor-specific synergy with HA1077. Since HA1077 induced the generation of L31H/Q/S substitution at relatively low frequency (3.4%), one possibility is that it has functional and/or direct interactions, if any, with these residues, likely with low affinity and specificity. However, this is unlikely the case because those variants were sensitive to HA1077. Our results showed that DCV and other NS5A inhibitors do not inhibit PRK2 activation (Supplementary Fig. 6), ruling out the possibility of their non-specific regulatory role in the PRK2 activation signaling pathway. DCV may target unknown cellular factors to exert its potent anti-HCV activity. Further studies are required to understand how HA1077, which inhibits both PRK2 and ROCK, displays synergy with DCV.

HCV NS5A phosphoprotein (447 amino acids) has three distinct structural domains: domain I (aa 1–213), domain II (aa 250–342), and domain III (aa 356–447). Domain I contains the N-terminal AH region acting as an NS5A membrane anchor (aa 5–25) and the Zn^2+^-binding domain, which are involved in NS5A dimerization and are required for HCV RNA replication^39,43–45^. Interestingly, we discovered a high polymorphism at L31 and its vicinity residues in the disordered linker region, which links the AH to the remaining part of the domain I (Fig. 6). The mutation frequency of 33.9–73.8% in the mice receiving DCV was much higher than the background, i.e., a silent mutation rate of 2.0% in saline-treated mice or an HA1077-induced substitution rate of 3.4%. These results clearly indicate that the high polymorphism at the PxxPxxP motif located within the NS5A domain I is not due to natural mutation propensity on this region. Indeed, deep-sequencing analysis of HCV full-genome in treatment-naïve individuals revealed that pre-existing RASs in HCV GT1a/b, GT2b, and GT3a are present at higher frequencies at various NS5A N-terminal residues (amino acid sites 30, 54, and 58) in the absence of DCV selection pressure^46^, rather than being enriched in the PxxPxxP motif as we observed in this study. Collectively, our results provide potential DCV-binding sites in the linker region between the N-terminal AH region and the domain Ia of NS5A, which carries the Zn^2+^-binding site. With this pool of multiple variants with diverse substitutions (~12 on L31) in this region, HCV might be equipped with adaptive power to conserve DCV-resistant variants for successful HCV rebound. According to predicted DCV-docking models, DCV binds to the NS5A dimer interfaces^39^. Therefore, such variants with diverse DCV RASs could form various sets of NS5A dimers to confer resistance to DCV and, thereby, to alter NS5A function in viral replication and virion assembly.

As shown with poliovirus and foot-and-mouth disease virus, mutants with lower fitness are supposed to be rapidly purged in large populations while selecting the most fitting progeny^47^. Despite the reduced replication capability of individual mutants with DCV RASs (Fig. 7), population of these mutants along with others constituted a greater proportion than wild-type HCV with NS5A L31 in the mice with undetectable serum HCV titers (Fig. 5a). These findings imply that the features of DCV-resistant HCV swarms might be different from those of individual mutants. Our results therefore reinforce the recent view on selection of viral quasispecies in which interconnected features of variant population contribute to the virus fitness through a process called group selection^48^. It would be of interest to test if genetic complementation occurred by co-infection of DCV RAVs to restore their replication competitiveness and consequently to maintain a large pool of DCV RAVs at sites of viral replication. One may speculate that HA1077 directly or indirectly suppresses such complementation effects to reduce mutation frequency in the mice that received DCV along with HA1077.

As discussed above, the pronounced diversity in the repertoire of substitutions on L31 and its neighboring residues was unexpected. In fact, previous studies, which were conducted with HCV replicons or using sera from patients showing HCV rebound or viral breakthrough, have not ever detected this high polymorphism on NS5A. This dramatic difference in NS5A polymorphism could be partly explained by the recent finding that DCV blocks the transfer of viral genome to assembly site^31^; therefore, only the abundant species targeted to the virus assembly sites are enriched in circulating virions. Supporting this hypothesis, we showed that the capability to produce infectious virus particles was impaired in the NS5A variants with L31/D/H/M/Q/S/V, F28S, or C92R substitution but not in the one bearing the Y93H substitution (Fig. 7e). Notably, the Y93H substitution in HCV GT1b also enhanced virus propagation^33,36^. Based on our results and previous findings on DCV RASs, it appears that the profile of DCV treatment-induced and pre-existing substitutions depends on HCV genotypes. Thus, our results underscore the importance of pre-screening patients with different HCV genotypes based on their own RAV profile information. More importantly, since those profiles seem to be remarkably different in infected cells and in secreted virions, our results also call for the importance of monitoring RAVs in liver tissues, even for those in patients with undetectable levels of HCV in sera to ensure successful management of HCV.

Currently, there is no clear consensus on which double or triple DAA combination is most effective in patients who experienced failure with DAA therapy^49^. For example, DCV/asunaprevir (ASV) treatment achieved extremely high SVR rates (~90%) in chronic HCV-GT1b infection^12^. DCV combination therapy was first approved in Japan with ASV^13,50^. However, this combination therapy generated DCV RAVs, with L31M or Y93F/H substitutions emerging in patients who failed to respond to the DCV/ASV therapy^13,50^. Kai *et al*.^51^ detected L31M/V-Y93H variants in patients with HCV GT1b who experienced virologic failure with DCV plus ASV. Kan *et al*.^52^ showed that DCV/ASV treatment is also ineffective in patients with simeprevir treatment failure. Therefore, additional clinical trial datasets are required to ensure competitiveness of the DCV plus ASV therapy as an IFN and ribavirin-free regimen. Recently, the United States Food and Drug Administration approved the DCV plus sofosbuvir combination therapy, which proved effective for patients with HCV GT1, 2, or 3, without administration of pegylated-IFN-α and ribavirin^53^. This DAA combination therapy is anticipated to limit the emergence of RAVs against both drugs. Currently, the combination of ledipasvir and sofosbuvir (also called Harvoni) is used for GT1 patients who did not experience SVR with IFN treatment^54^. Sofosbuvir/velpatasvir/voxilaprevir (Vosevi; Gilead), which was approved in July 2017, is a certainly one of the most advanced DAA combination regimens. In 2017, glecaprevir and pibrentasvir (Maviret; AbbVie) combination was also approved in Europe for treatment of all genotypes. These and other DAA combinations in clinical trials^4^ will provide multiple treatment options for patients who received unsuccessful treatment with DAA-based regimens. In the phase 3 trial^55^, Vosevi was effective in 97% enrolled patients. However, RASs in the NS5A were found in 2 patients even though they only reported RASs that were present in more than 15% of sequence reads. Further monitoring is thus needed to ensure that this triple combination therapy is effective for patients in whom treatment with diverse NS5A inhibitors had previously failed.

Despite multiple DAA combination options, there is no curative therapy option using HTAs. We showed that the frequency of DCV-resistant mutations on both L31 and Y93 can be substantially decreased by HA1077 combination, and that HA1077 improves the efficacy of DCV. However, the dose and schedule used in this study did not result in complete prevention of HCV rebound by combination of DCV and HA1077; HCV titers remained below the detectable limit on day 12 or 15 till day 24, at which DCV RAVs emerged. This might be due to the presence of unprecedented diversity of NS5A polymorphism induced by DCV during HCV propagation in the immunocompromised mice. Host immunity including innate and adaptive immune responses is a potent driving force in viral evolution^56^. It remains to be determined whether an unexpected high evolvability of HCV NS5A gene, which we observed in DCV-receiving NOD-SCID mice, also occurs in immunocompetent hosts. Further studies are also required to test whether HCV with DCV RASs can be controlled more effectively by using improved PRK2 inhibitors.

In summary, we provide proof of concept for the inclusion of an HTA in DAA therapies by demonstrating the synergistic efficacy of HA1077 plus DCV combination. This HTA/DAA combination therapy might be an attractive strategy for better management of patients who carry pre-existing resistance variants or have emerging DCV RAVs. However, caution should be exercised on the use of this potentially promising combination therapy in patients across all age groups because it might be associated with side effects caused by drug-drug interactions or by inhibiting the activity of the host kinases PRK2 and ROCK.

## Methods

### Reagents: HCV inhibitors, siRNAs, staining reagents, and antibodies

Asunaprevir, boceprevir, dasabuvir, and mericitabine were purchased from MedChem Express (Monmouth Junction, NJ, USA). Telaprevir, ledipasvir, and daclatasvir were purchased from Selleckchem (Houston, TX, USA). Ombitasvir was purchased from Clearsynth (Lotus Business Park, Andheri West, India). Simeprevir was obtained from ApexBio (Boston, MA, USA). The PRK2 inhibitor HA1077 was purchased from Sigma-Aldrich (Saint Louis, MO, USA) or Selleckchem. The siRNAs used for depletion of PRK2, ROCK1, and ROCK2 and the scrambled control siRNA (siCtrl) were described previously^21^. siRNAs were transfected into cells using Lipofectamine RNAiMAX (Invitrogen, CA, USA). PKH67 dye was obtained from Sigma-Aldrich and used for HCV particle labeling according to the manufacturer’s protocol. The following antibodies were used: a mouse monoclonal anti-HCV core antibody (clone C7–50) from Abcam (Cambridge, UK); rabbit polyclonal anti-PRK2 and p-PRK2(Thr816) antibodies from Cell Signaling Technology (Danvers, MA, USA); goat polyclonal anti-pPRK2(Thr816), and mouse monoclonal anti-ROCK1 (4H247) and ROCK2 (30-J) antibodies from Santa Cruz Biotechnology (Santa Cruz, CA, USA); and Alexa fluor488 goat anti-mouse IgG antibody from Invitrogen.

### Cell culture and virus infection

The human hepatocellular carcinoma cell line Huh7 was grown in Dulbecco’s modified Eagle’s medium (DMEM) supplemented with 10% fetal bovine serum, 2 mM L-glutamine, 100 U/ml of penicillin, 100 μg/ml of streptomycin, and 0.1 mM of nonessential amino acids under standard culture conditions (5% CO_2_, 37 °C). The Huh7-derived cell line R-1, which harbors a GT1b subgenomic replicon^22,57^, was maintained in DMEM with 1 mg/ml of G418. The infectious cDNA clone of GT2a HCV JFH1^58^, which was linearized with Xba1 and treated with Mung bean nuclease (Takara, Kyoto, Japan), was used as a template for the preparation of viral RNA transcripts by using the T7 MEGAscript kit (Ambion, Austin, TX, USA). The JFH1 RNA was electroporated into Huh7 cells. After 24 h, medium was changed to fresh medium prior to the collection of the virus in culture supernatant at 3 days post-transfection. The culture medium was cleared by low-speed centrifugation at ~900 × *g* for 5 min and passed through a Millipore filter (20-nm pore size) to obtain cell culture-infectious HCV (HCVcc). For infection, Huh7 cells were incubated with HCVcc at a multiplicity of infection of 0.25–0.5 with periodic rocking. Cells were then washed three times with phosphate-buffered saline (PBS) and maintained in complete DMEM.

### Reverse transcription-quantitative PCR

Total RNA was extracted from R-1 cells, or HCV (JFH1)-infected or HCV RNA-transfected Huh7 cells using Trizol reagent (Invitrogen, Carlsbad, CA, USA). Real-time reverse transcription-quantitative PCR (RT-qPCR) for the determination of HCV RNA titer was performed using specific primers for GT1b or 2a HCV, TaqMan probe targeting HCV 5′-UTR region, and iQ Supermix (Bio-Rad, Herules, CA). The primers used for RT-qPCR are as follows: sense primer 5′-UTR F (5′-GCGTCTAGCCATGGCCTTAGTATGAGTGTC-3′) and antisense primer 5′-UTR R (5′-ACCACAAGGCCTTTCGCGACCCAACACTAC-3′) for GT1b; sense primer 5′-UTR F (5′-CGGGAGAGCCATAGTGG-3′) and antisense primer 5′-UTR R (5′- AGTACCACAAGGCCTTTCG-3′) for GT2a; and a dual fluorophore-labeled probe [5′-FAM (6-carboxyfluorescein)-CTGCGGAACCGGTGAGTACAC-TAMRA (6-carboxytetramethylrhodamine)-3′].

### Analysis of the synergistic effect

The combination index (CI) of HA1077 and HCV inhibitor combinations was calculated using the CompuSyn software (version 3.0.1; CompuSyn, Inc., Paramus, NJ, USA). The combination index (CI) value, calculated by the multiple drug effect equation, was used to estimate the drug combination effects: synergism, CI < 1; additive effect, CI = 1; and antagonism, CI > 1. The three-dimensional (3D) surface plot of CI values was drawn using the Number Cruncher Statistical System (NCSS) software (version 11, NCSS, Kaysville, UT, USA).

### Site-directed mutagenesis

The resistance-associated NS5A mutations were introduced into the JFH1 infectious clone by site-directed mutation using the QuikChange II XL site-directed mutagenesis kit (Agilent Technologies, Santa Clara, CA, USA). Primers used for mutagenesis are listed in Supplementary Table 1.

### Northern blotting

Total RNA (5 μg), which was extracted from Huh7 cells and JFH1 RNA-transfected Huh7 cells using Trizol reagent (Invitrogen), was resolved on a denaturing agarose (0.7%) gel and transferred onto a positive charged nylon membrane (Roche Diagnostics, Mannheim, Germany) and fixed to the membrane by UV-crosslinking. HCV (JFH1) and GAPDH probes were generated by random labeling of HCV and glyceraldehyde 3-phosphate dehydrogenase (GAPDH) cDNA fragments prepared by RT-PCR using the following primer sets: sense primer JFH1 F (5′-CCAACCTGCTCATGGAGG-3′), antisense primer JFH1 R (5′-TCCACGCAGAACACCTCA-3′), sense primer GAPDH F (5′-GAAGGTGAAGGTCGGAGTC-3′), and antisense primer GAPDH R (5′-GGGACTCCCCAGCAGTG-3′). The PCR-amplified DNA fragments were radiolabeled using [α-^32^P] dCTP and Rediprime II DNA labeling system (Amersham Biosciences, PA, USA). Membranes were pre-hybridized for 30 min and then hybridized with HCV-specific radiolabeled probes overnight. After washing, membranes were quantified using PhosphorImager and normalized to GAPDH. Uncropped images of northern blots are provided in Supplementary Fig. 7b.

### Immunoblotting and immunocytochemistry

Immunoblotting analysis was carried out as described previously^21^. Uncropped images of immunoblots are provided in Supplementary Fig. 7a,c,d. Huh7 cells grown on Lab-Tek 4-well chamber slides (Nunc, Saint Louis, MO, USA) were infected with JFH1 HCVcc when the cells were approximately 50% confluent. The HCVcc was obtained from the culture supernatant of JFH1 RNA-transfected Huh7 cells at 3 days post-transfection. Two days after infection using culture supernatants concentrated 50-fold in a Centricon (MW cut-off of 10,000; Amicon, Danvers, MA, USA), cells were fixed with 4% paraformaldehyde in methanol for 15 min at room temperature, washed three times with cold PBS, then permeabilized with PBS containing 0.2% Triton X-100 for 10 min at room temperature. After washing three times with PBS, the cells were treated with a blocking solution (3% horse serum in PBS) for 30 min at room temperature. Cells were further incubated with a monoclonal anti-HCV core antibody overnight at 4 °C, then washed three times with PBS. Cells were further incubated with fluorescent-conjugated secondary antibodies (Invitrogen) for 2 h at room temperature to visualize the core antigen, and then washed three times with PBS. The HCV Core-positive area among 50 total cells from three confocal images was quantified using ImageJ program (https://imagej.nih.gov/ij/). Immunostaining for HCV core antigen in the xenograft tissues was described previously^21^. Nuclei were stained with 1 µM 4**′**,6**′**-diamidino-2-phenylindole (DAPI) in PBS. Fluorescent signal was visualized using a confocal laser scanning microscope (Zeiss LSM 700 META, Carl Zeiss, Oberkochen, Germany).

### Sequencing analysis and bioinformatics

Total RNA (5 μg), which was extracted from 5-mm cube xenografts retrieved from mice using Trizol, was used for the synthesis of HCV NS5A cDNA [nt 6,339–6,560; nucleotide positions are numbered according to the JFH1 HCV GT2a sequence (GenBank accession no. AB047639)] using a reverse primer (5′-GACTGGAGTTCAGACGTGTGCTCTTCCGATCTGGCGCGCACTGGCCCTCCGT-3′). The cDNA was PCR**-**amplified using a forward primer (5′-AATGATACGGCGACCACCGAGATCTACACTCTTTCCCTACACGACGCTCTTCCGATCTCCTCTAAATTGTTCCCCAAG-3′) and reverse primers tagged with the Illumina sequencing adapters. The resulting amplicons were sequenced on the MiSeq benchtop sequencer (Illumina, San Diego, CA, USA). After removal of Illumina adapter sequences using FASTX-toolkit (http://hannonlab.cshl.edu/fastx_tollkit/), reads were analyzed by the Visual Basic Application (VBA)-coded macro running in the Microsoft Excel environment. The cutoff threshold for detecting an amino acid substitution frequency was 1% to account for RT-PCR and sequencing errors.

### Animal experiments

All animal experiments were performed in accordance with the Korean Food and Drug Administration guidelines. Protocols were reviewed and approved by the Institutional Animal Care and Use Committee of Yonsei University (Permit No: IACUC-A-201506-332-02 and IACUC-A-201606-231-02). At the termination of experiments, all mice were euthanized by CO_2_ inhalation. To evaluate *in vivo* efficacy of antiviral agents on HCV, NOD/SCID mice bearing HCV-replicating Huh7 xenografts were used^21^. Briefly, HCV RNA-transfected cells mixed with Matrigel were injected into the large lobes of the livers of anesthetized immunodeficient NOD/SCID male mice (5 weeks of age, 18–20 g body weight). Four weeks after implantation, compounds dissolved in saline were orally administered to mice using a feeding needle (Fuchigami, Japan). Serum HCV titer was monitored by RT-qPCR.

### Statistical analysis

Statistical analyses were performed using GraphPad Prism (version 6.01; GraphPad Software Inc., La Jolla, CA, USA). Results are presented as mean ± standard deviation (s.d.) from three independent experiments unless otherwise specified. Specific tests used to determine statistical difference are noted in each figure legend. *P* values less than 0.05 were considered statistically significant.

## Electronic supplementary material

Supplementary Information

## Data Availability

The authors declare that all other relevant data are available from the authors upon reasonable request.

## References

[1] Thomas DL (2013). Global control of hepatitis C: where challenge meets opportunity. Nat. Med..

[2] Tai CL, Chi WK, Chen DS, Hwang LH (1996). The helicase activity associated with hepatitis C virus nonstructural protein 3 (NS3). J. Virol..

[3] Pawlotsky JM (2014). New hepatitis C therapies: the toolbox, strategies, and challenges. Gastroenterology.

[4] Li G, De Clercq E (2017). Current therapy for chronic hepatitis C: The role of direct-acting antivirals. Antiviral Res..

[5] De Clercq E, Li G (2016). Approved antiviral drugs over the past 50 years. Clin. Microbiol. Rev..

[6] Smith MA, Regal RE, Mohammad RA (2016). Daclatasvir: a NS5A replication complex inhibitor for hepatitis C infection. Ann. Pharmacother..

[7] McCown MF (2008). The hepatitis C virus replicon presents a higher barrier to resistance to nucleoside analogs than to nonnucleoside polymerase or protease inhibitors. Antimicrob. Agents Chemother..

[8] Geller R (2016). Highly heterogeneous mutation rates in the hepatitis C virus genome. Nat. Microbiol..

[9] Sarrazin C, Zeuzem S (2010). Resistance to direct antiviral agents in patients with hepatitis C virus infection. Gastroenterology.

[10] O’Boyle DR (2015). Synergistic activity of combined NS5A inhibitors. Antimicrob. Agents Chemother..

[11] Pelosi LA, Voss S, Liu M, Gao M, Lemm JA (2012). Effect on hepatitis C virus replication of combinations of direct-acting antivirals, including NS5A inhibitor daclatasvir. Antimicrob. Agents Chemother..

[12] Chayama K (2012). Dual therapy with the nonstructural protein 5A inhibitor, daclatasvir, and the nonstructural protein 3 protease inhibitor, asunaprevir, in hepatitis C virus genotype 1b-infected null responders. Hepatology.

[13] Yoshimi S (2015). Long term persistence of NS5A inhibitor-resistant hepatitis C virus in patients who failed daclatasvir and asunaprevir therapy. J. Med. Virol..

[14] McPhee F (2013). Resistance analysis of hepatitis C virus genotype 1 prior treatment null responders receiving daclatasvir and asunaprevir. Hepatology.

[15] Koizumi Y (2017). Quantifying antiviral activity optimizes drug combinations against hepatitis C virus infection. Proc. Natl. Acad. Sci. USA.

[16] Bartenschlager R, Lohmann V, Penin F (2013). The molecular and structural basis of advanced antiviral therapy for hepatitis C virus infection. Nat. Rev. Microbiol..

[17] Hopkins S (2012). The cyclophilin inhibitor SCY-635 suppresses viral replication and induces endogenous interferons in patients with chronic HCV genotype 1 infection. J. Hepatol..

[18] Janssen HL (2013). Treatment of HCV infection by targeting microRNA. N. Engl. J. Med..

[19] Kim SJ, Kim JH, Kim YG, Lim HS, Oh JW (2004). Protein kinase C-related kinase 2 regulates hepatitis C virus RNA polymerase function by phosphorylation. J. Biol. Chem..

[20] Han SH (2014). Phosphorylation of hepatitis C virus RNA polymerases ser29 and ser42 by protein kinase C-related kinase 2 regulates viral RNA replication. J. Virol..

[21] Moon JS (2016). Inhibition of hepatitis C virus in mouse models by lipidoid nanoparticle-mediated systemic delivery of siRNA against PRK2. Nanomedicine: Nanotechnology, Biology, and Medicine.

[22] Kim SJ, Kim JH, Sun JM, Kim MG, Oh JW (2009). Suppression of hepatitis C virus replication by protein kinase C-related kinase 2 inhibitors that block phosphorylation of viral RNA polymerase. J..Viral. Hepat..

[23] Davies SP, Reddy H, Caivano M, Cohen P (2000). Specificity and mechanism of action of some commonly used protein kinase inhibitors. Biochem. J..

[24] Sasaki Y, Suzuki M, Hidaka H (2002). The novel and specific Rho-kinase inhibitor (S)-(+)-2-methyl-1-[(4-methyl-5-isoquinoline)sulfonyl]-homopiperazine as a probing molecule for Rho-kinase-involved pathway. Pharmacol. Ther..

[25] Shibuya M, Hirai S, Seto M, Satoh S, Ohtomo E (2005). Effects of fasudil in acute ischemic stroke: results of a prospective placebo-controlled double-blind trial. J. Neurol. Sci..

[26] Masumoto A (2002). Suppression of coronary artery spasm by the Rho-kinase inhibitor fasudil in patients with vasospastic angina. Circulation.

[27] Ying H (2006). The Rho kinase inhibitor fasudil inhibits tumor progression in human and rat tumor models. Mol. Cancer Ther..

[28] Li Q (2014). Integrative functional genomics of hepatitis C virus infection identifies host dependencies in complete viral replication cycle. PLoS Pathog..

[29] Eyre NS, Beard MR (2014). HCV NS5A inhibitors disrupt replication factory formation: a novel mechanism of antiviral action. Gastroenterology.

[30] Berger C (2014). Daclatasvir-like inhibitors of NS5A block early biogenesis of hepatitis C virus-induced membranous replication factories, independent of RNA replication. Gastroenterology.

[31] Boson B (2017). Daclatasvir prevents hepatitis C virus infectivity by blocking transfer of the viral genome to assembly sites. Gastroenterology.

[32] Gao M (2010). Chemical genetics strategy identifies an HCV NS5A inhibitor with a potent clinical effect. Nature.

[33] Fridell RA, Qiu D, Wang C, Valera L, Gao M (2010). Resistance analysis of the hepatitis C virus NS5A inhibitor BMS-790052 in an *in vitro* replicon system. Antimicrob. Agents Chemother..

[34] Wang C (2014). Comparison of daclatasvir resistance barriers on NS5A from hepatitis C virus genotypes 1 to 6: implications for cross-genotype activity. Antimicrob. Agents Chemother..

[35] Fridell RA (2011). Distinct functions of NS5A in hepatitis C virus RNA replication uncovered by studies with the NS5A inhibitor BMS-790052. J. Virol..

[36] Nitta S (2016). Effects of resistance-associated NS5A mutations in hepatitis C virus on viral production and susceptibility to antiviral reagents. Sci. Rep..

[37] Ahmed M, Pal A, Houghton M, Barakat K (2016). A comprehensive computational analysis for the binding modes of hepatitis C virus NS5A inhibitors: the question of symmetry. ACS Infect. Dis..

[38] Penin F (2004). Structure and function of the membrane anchor domain of hepatitis C virus nonstructural protein 5A. J. Biol. Chem..

[39] Nettles JH (2014). Asymmetric binding to NS5A by daclatasvir (BMS-790052) and analogs suggests two novel modes of HCV inhibition. J. Med. Chem..

[40] Issur M, Gotte M (2014). Resistance patterns associated with HCV NS5A inhibitors provide limited insight into drug binding. Viruses.

[41] Kim S, Welsch C, Yi M, Lemon SM (2011). Regulation of the production of infectious genotype 1a hepatitis C virus by NS5A domain III. J. Virol..

[42] Reiss S (2011). Recruitment and activation of a lipid kinase by hepatitis C virus NS5A is essential for integrity of the membranous replication compartment. Cell Host Microbe.

[43] Brass V (2002). An amino-terminal amphipathic alpha-helix mediates membrane association of the hepatitis C virus nonstructural protein 5A. J. Biol. Chem..

[44] Tellinghuisen TL, Marcotrigiano J, Gorbalenya AE, Rice CM (2004). The NS5A protein of hepatitis C virus is a zinc metalloprotein. J. Biol. Chem..

[45] Tellinghuisen TL, Marcotrigiano J, Rice CM (2005). Structure of the zinc-binding domain of an essential component of the hepatitis C virus replicase. Nature.

[46] Eltahla AA (2017). Dynamic evolution of hepatitis C virus resistance-associated substitutions in the absence of antiviral treatment. Sci. Rep..

[47] Lauring AS, Frydman J, Andino R (2013). The role of mutational robustness in RNA virus evolution. Nat. Rev. Microbiol..

[48] Andino R, Domingo E (2015). Viral quasispecies. Virology.

[49] Lau G (2016). Efficacy and safety of 3-week response-guided triple direct-acting antiviral therapy for chronic hepatitis C infection: a phase 2, open-label, proof-of-concept study. Lancet Gastroenterol Hepatol..

[50] Poveda E (2014). Update on hepatitis C virus resistance to direct-acting antiviral agents. Antiviral Res..

[51] Kai Y (2017). Baseline quasispecies selection and novel mutations contribute to emerging resistance-associated substitutions in hepatitis C virus after direct-acting antiviral treatment. Sci. Rep..

[52] Kan H (2016). Protease inhibitor resistance remains even after mutant strains become undetectable by deep sequencing. J. Infect. Dis..

[53] Sulkowski MS (2014). Daclatasvir plus sofosbuvir for previously treated or untreated chronic HCV infection. N. Engl. J. Med..

[54] Afdhal N (2014). Ledipasvir and sofosbuvir for previously treated HCV genotype 1infection. New. Engl. J. Med..

[55] Bourliere M (2017). Sofosbuvir, velpatasvir, and voxilaprevir for previously treated HCV infection. N. Engl. J. Med..

[56] Poirier EZ, Vignuzzi M (2017). Virus population dynamics during infection. Curr. Opin. Virol..

[57] Zhu Q, Guo JT, Seeger C (2003). Replication of hepatitis C virus subgenomes in nonhepatic epithelial and mouse hepatoma cells. J. Virol..

[58] Wakita T (2005). Production of infectious hepatitis C virus in tissue culture from a cloned viral genome. Nat. Med..

