# Supplementary material for: HA1077 displays synergistic activity with daclatasvir against hepatitis C virus and suppresses the emergence of NS5A resistance-associated substitutions in mice
Source: Sci Rep. 2018 Aug 20;8:12469. doi: 10.1038/s41598-018-30460-3 (PMC6102265; doi:10.1038/s41598-018-30460-3)
Supplement: Supplementary file 1 — Supplementary Information [file 41598_2018_30460_MOESM1_ESM.pdf]

Supplementary Information

HA1077 displays synergistic activity with daclatasvir against hepatitis C virus and suppresses the emergence of NS5A resistance-associated substitutions in mice

Seung-Hoon Lee, Jae-Su Moon, Bo-Yeong Pak, Geon-Woo Kim, Wooseong Lee, Hee Cho, SangKyu Kim, Seong-Jun Kim and Jong-Won Oh*

Contents:

Supplementary Figures 1–7
Supplementary Table 1

Correspondence should be addressed to J.-W.O. (jwoh@yonsei.ac.kr).

Supplementary Figures

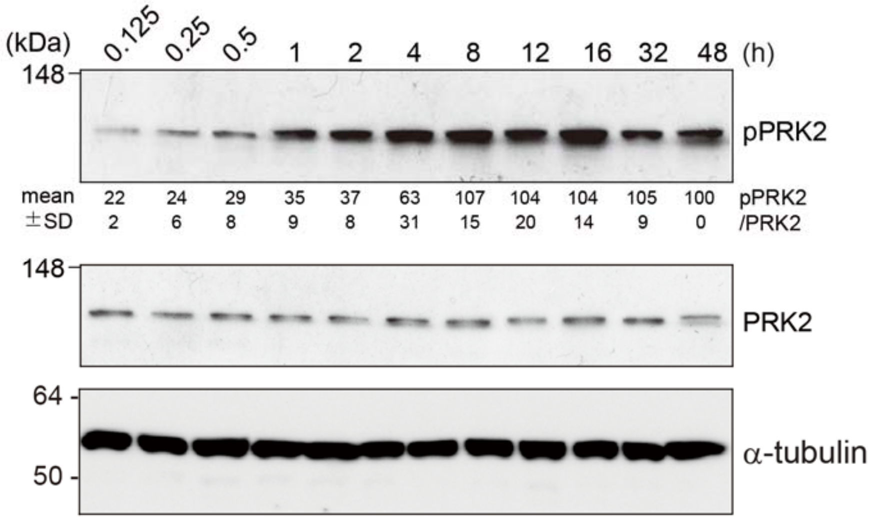

Supplementary Figure 1. Time course of PRK2 activation inhibition by HA1077. Immunoblotting analysis for the indicated proteins in Huh7 cells treated with HA1077 for the indicated times. The immunoblotting images shown are from one representative experiment of three independent experiments with similar results. Relative signals (% of pPRK2/PRK2 at time 48 h), which were determined by densitometric analysis of pPRK2 and PRK2 levels, are shown below a representative blot.

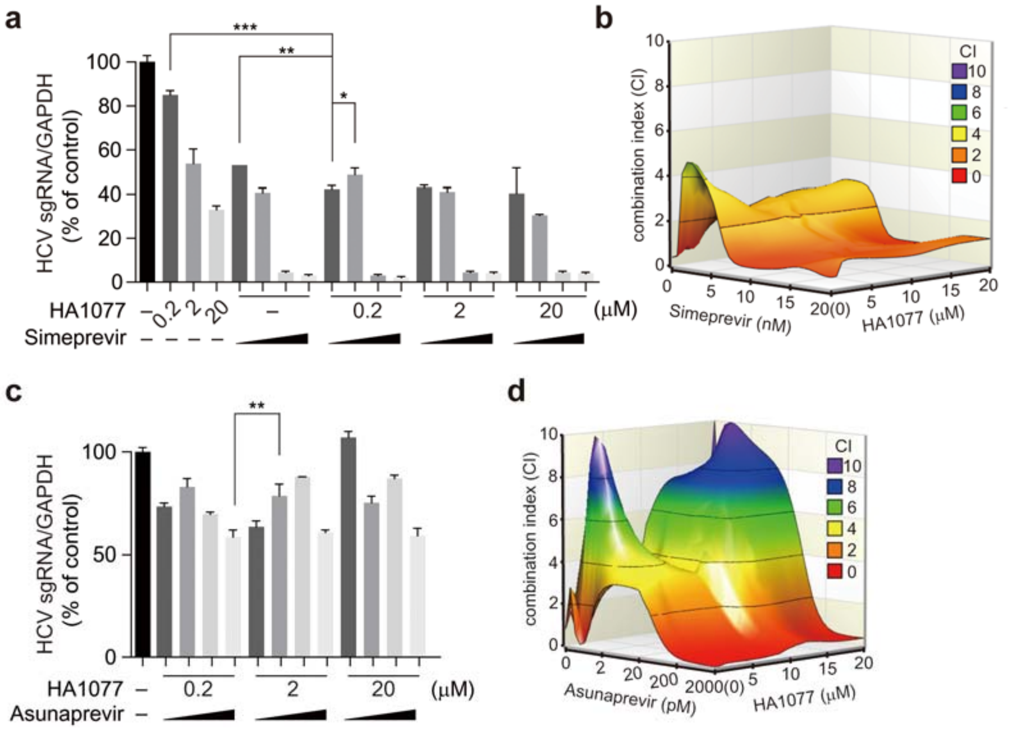

Supplementary Figure 2. Anti-HCV efficacy of HA1077 plus NS3/4A protease inhibitor combinations. R-1 cells were treated for 48 h with increasing concentrations of HA1077 in the absence or presence of simeprevir (**a**,**b**; 200 pM, 2 nM, 10 nM, and 20 nM) or asunaprevir (**c**,**d**; 2 pM, 20 pM, 200 pM, and 2 nM). HCV RNA levels were determined by RT-qPCR. The concentrations of the NS3/4A protease inhibitor simeprevir and asunaprevir used in the assay were based on the EC_{50} of each compound on HCV GT1b[55,56], which was 8 nM and 1.5 nM, respectively. In (**b**,**d**), shown are the combination index (CI) values determined as described in Fig. 3. **P* < 0.05; ***P* < 0.01; ****P* < 0.001; by unpaired two-tailed *t*-test.

—Both HA1077 and simeprevir dose-dependently inhibited HCV replication (**a**). Most of the combinations of HA1077 and simeprevir tested in this study did not show substantial synergistic effects (**b**). Simeprevir displayed a marginal, but statistically significant, synergy in lowering HCV loads at the lowest tested concentration (0.2 nM) when combined with 0.2 μM HA1077; however, 2 nM simeprevir slightly increased HCV titer). Similarly, almost no synergistic effect was observed with various combinations of asunaprevir and HA1077 (**c**). Some combinations of these compounds (2 μM HA1077 and 20 pM or 200 pM asunaprevir) showed antagonistic effects, while their synergy was only observed when higher doses (200 pM and 2 nM) of asunaprevir were used with various concentrations of HA1077 (**d**).

Furthermore, with the two first-generation DAAs boceprevir and telaprevir, HA1077 did not synergistically inhibit HCV replication in R-1 cells. For instance, 20 μM HA1077 administered with 200 nM boceprevir or 500 nM telaprevir did not enhance or suppress antiviral activity of each DAA compound. Altogether, these results suggest that combinations of HA1077 and the NS3/4A protease inhibitors tested in this study have, if any, only marginal or dose-independent synergy or additive effects at a very limited range of the tested concentrations.

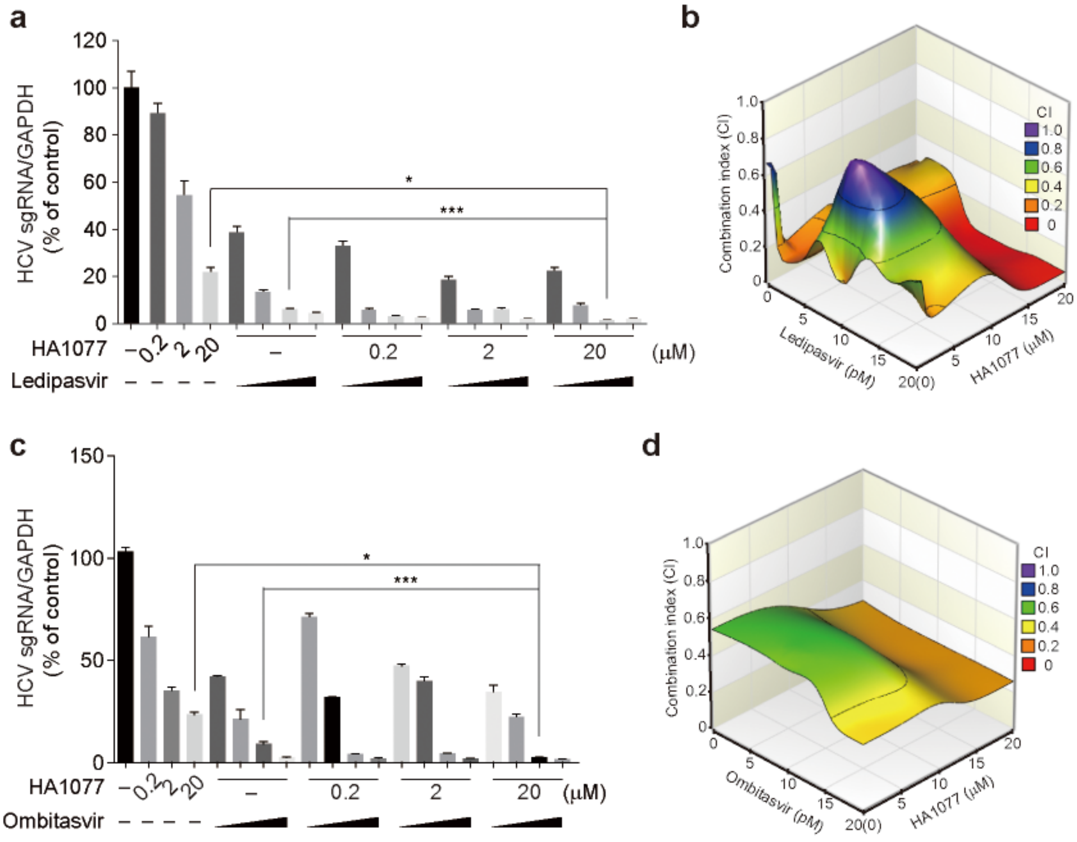

Supplementary Figure 3. Synergistic inhibition of HCV by HA1077 plus NS5A inhibitor combinations. (**a**,**c**) Anti-HCV efficacy of various combinations of HA1077 (0, 0.2, 2, and 20 µM) and the NS5A inhibitor ledipasvir (0, 200 fM, 2 pM, 10 pM, and 20 pM) or ombitasvir (0, 200 fM, 2 pM, 10 pM, and 20 pM) was tested in R-1 cells. Two days after drug treatment, HCV RNA level was determined by RT-qPCR. (**b**,**d**) CI values for the combination therapies were determined as described in Fig. 3. **P* < 0.05; ****P* ≤ 0.001; by unpaired two-tailed Student's *t*-test.

—Compared with HA1077 alone treatment, its combination with ledipasvir reduced HCV RNA levels dramatically, with an almost 2 $\log_{10}$-reduction by treatment with 20 µM HA1077 plus 10 pM ledipasvir (**a**). The 3D plot analysis of the predicted CI values revealed that all combinations of these two compounds tested show a synergistic anti-HCV effect (**b**). Ombitasvir, which inhibited HCV replication dose dependently as expected when used alone, also lowered HCV RNA levels synergistically with HA1077. Higher concentrations of ombitasvir (10 pM and 20 pM) synergistically reduced HCV RNA titer in cells co-treated with HA1077 at various concentrations (**c**,**d**).

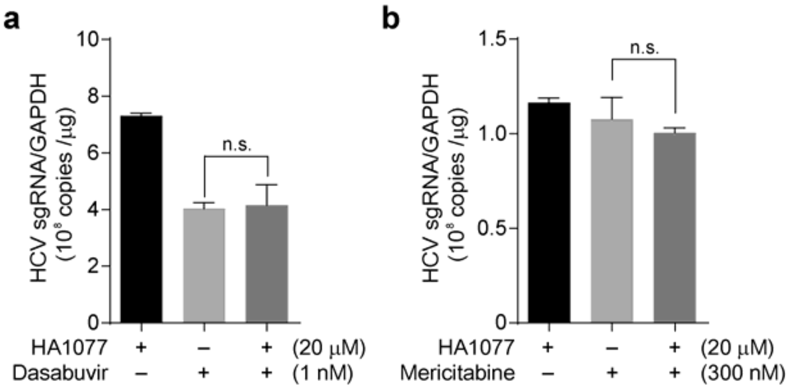

Supplementary Figure 4. No synergistic inhibition of HCV by HA1077 plus NS5B inhibitor combinations. HCV RNA titer in R-1 cells treated with combination of HA1077 and dasabuvir (**a**) or mericitabine (**b**) for 48 h was determined by RT-qPCR. Data were from two independent experiments, each involving triplicates. n.s., not significant; by unpaired two-tailed Student's *t*-test.

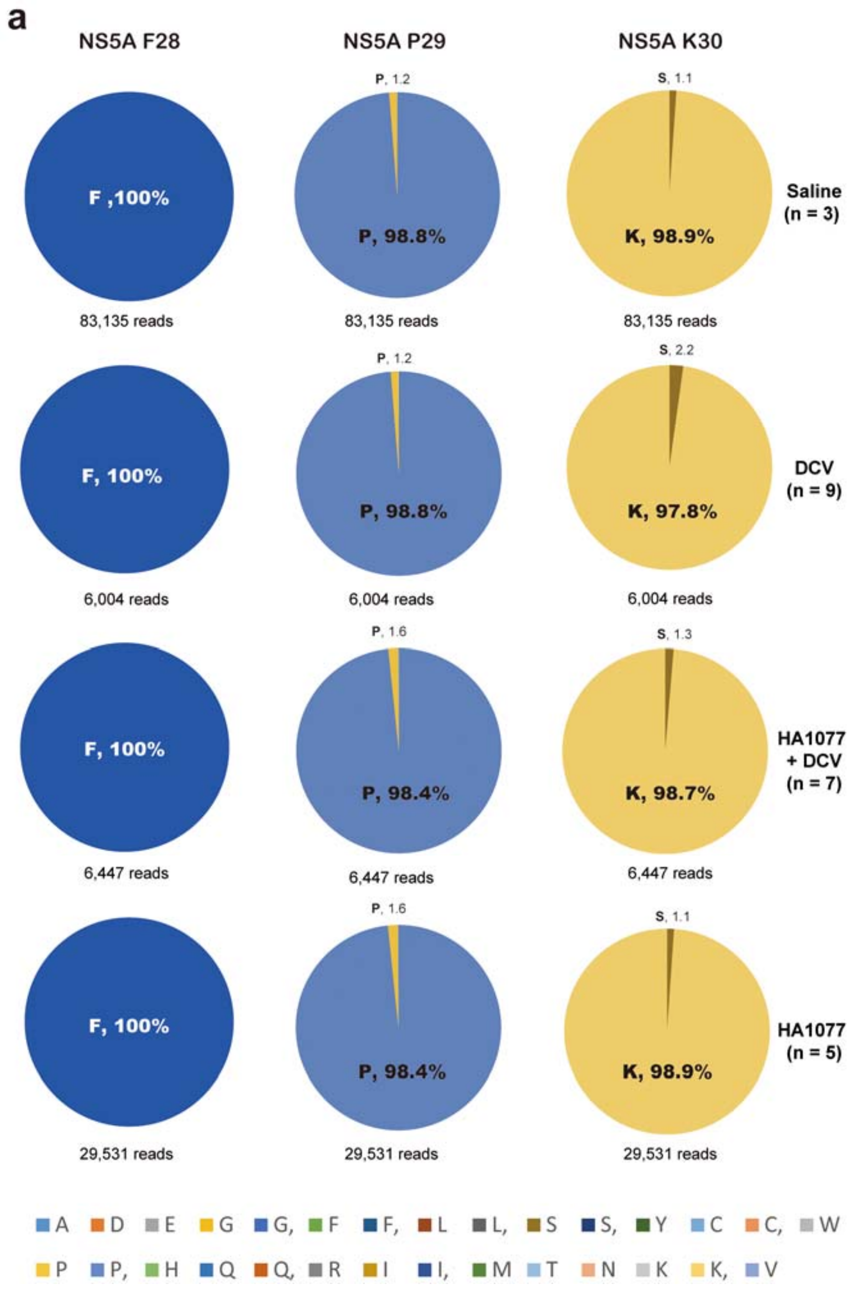

Supplementary Figure 5. DCV-induced polymorphism at the NS5A N-terminal region spanning from F28 to Q40 and at the C92 known to confer resistance to DCV in HCV GT2a. HCV (JFH1)-replicating Huh7 xenografts recovered from the mice receiving saline or the indicated drug(s) for 27 days as described in Fig. 4a were used for analysis of DCV resistance-associated mutations on the residue F28–K30 (**a**), P32/G33 (**b**), L34/P35 (**c**), F36/I37 (**d**), S38/C39 (**e**), and Q40/C92 (**f**), as in Fig. 5a.

– continued next page

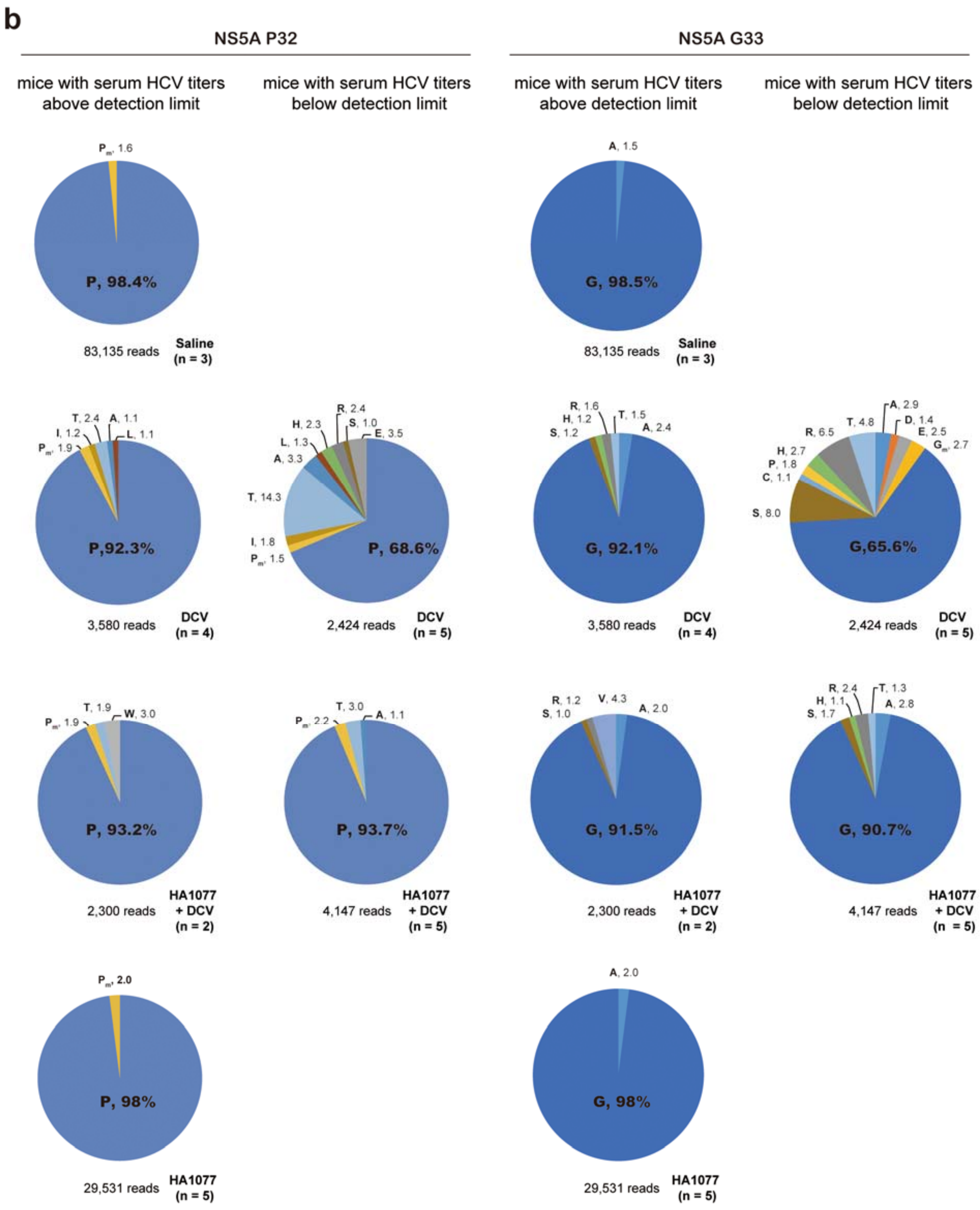

– continued next page

c

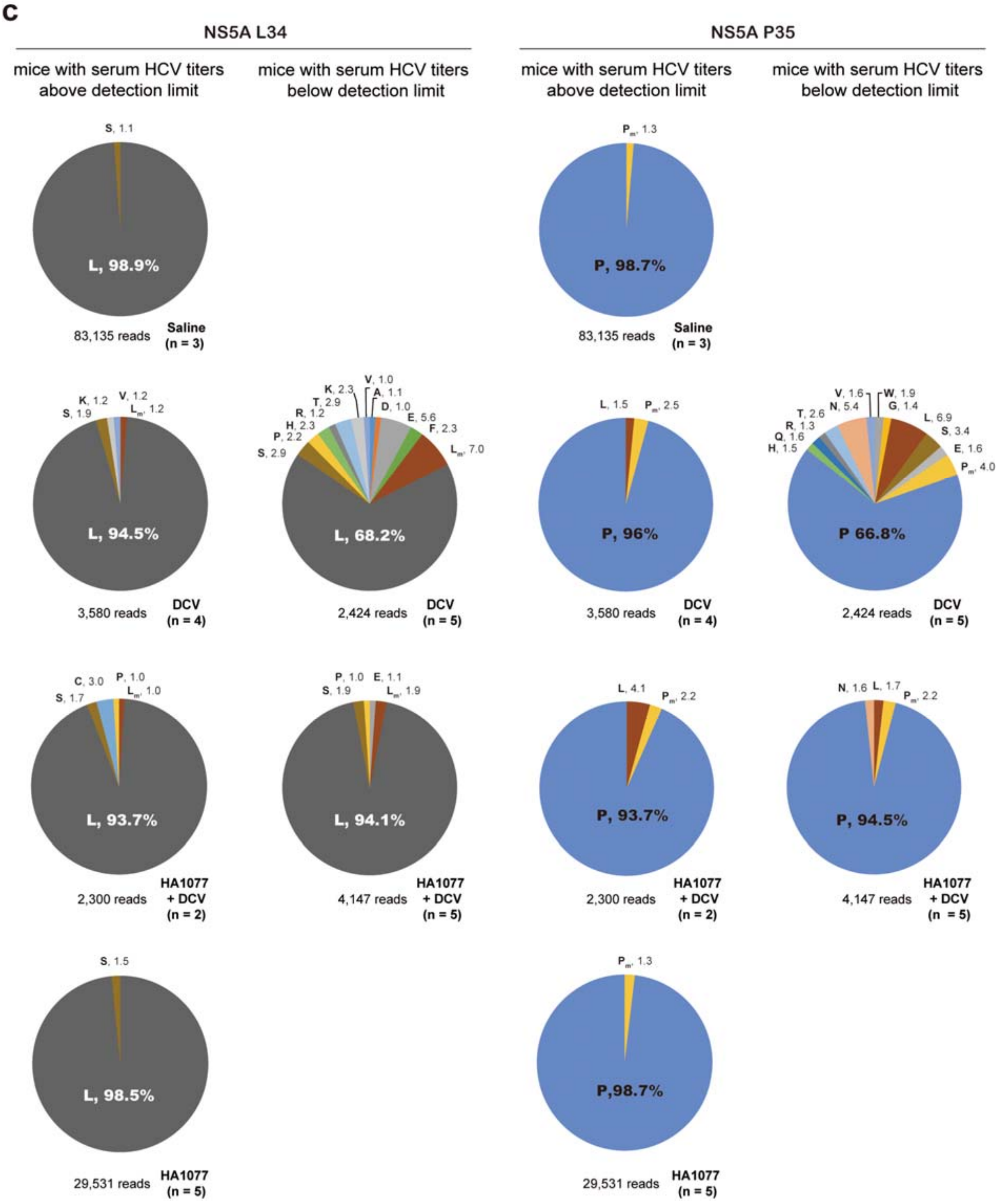

– continued next page

d

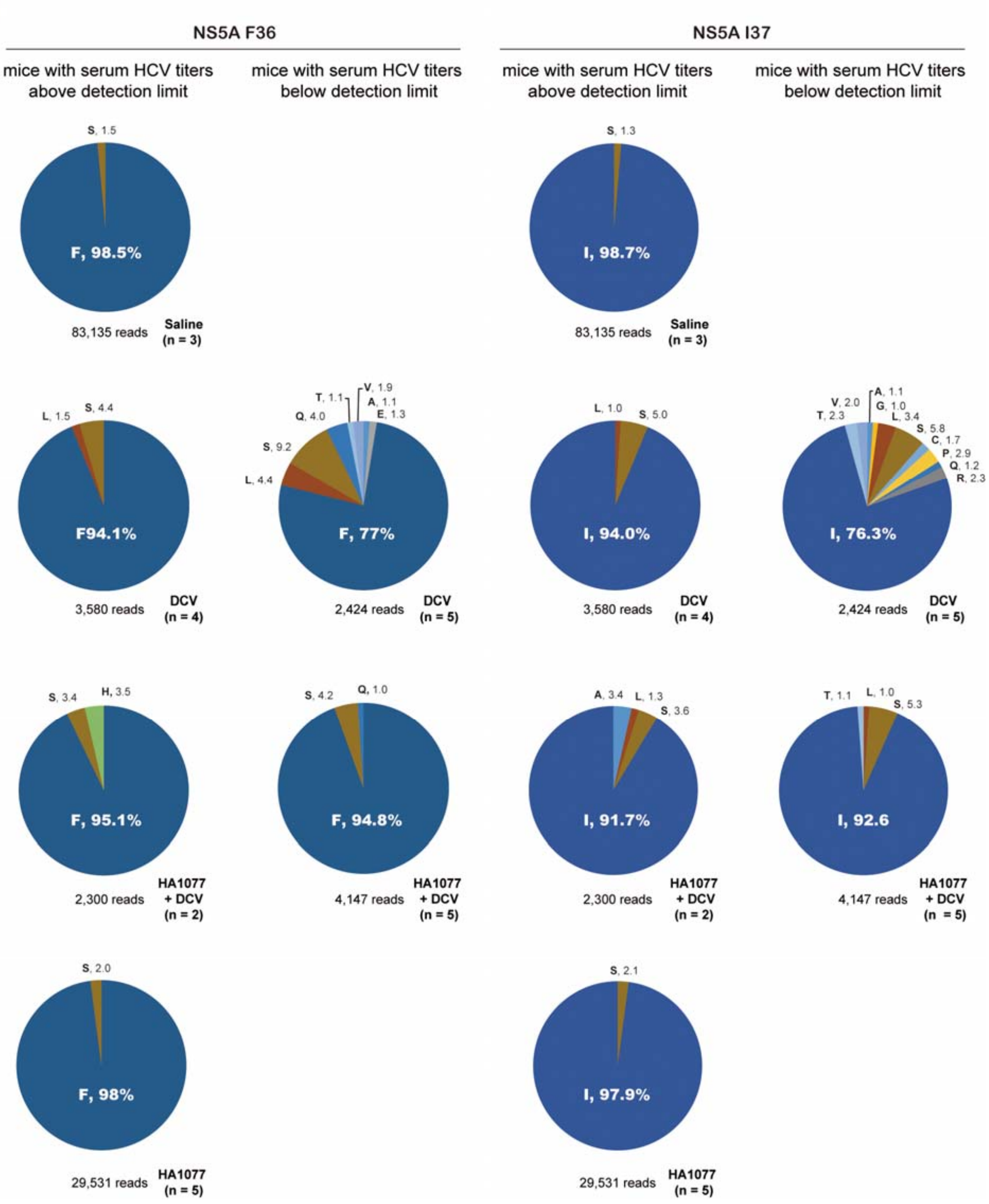

– continued next page

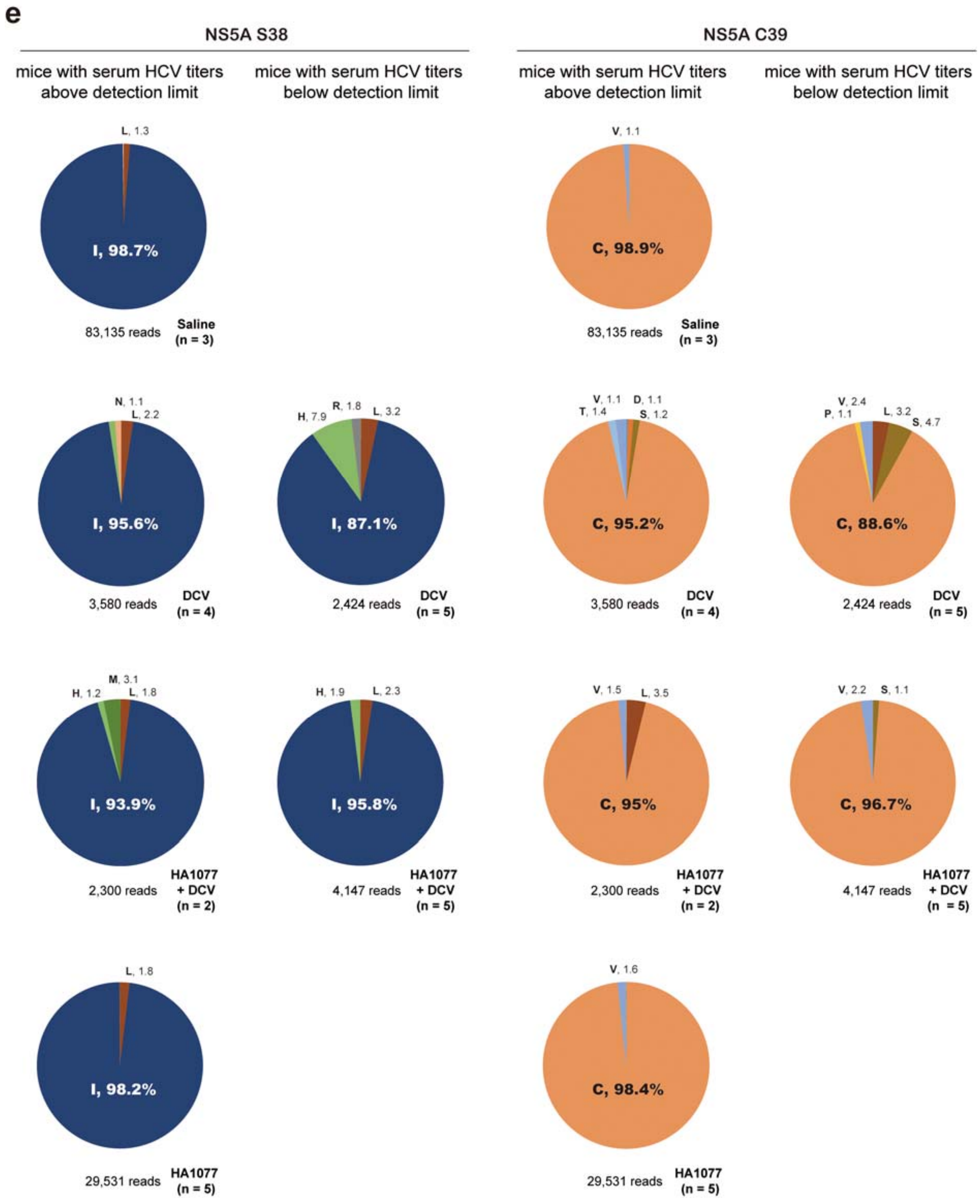

– continued next page

f

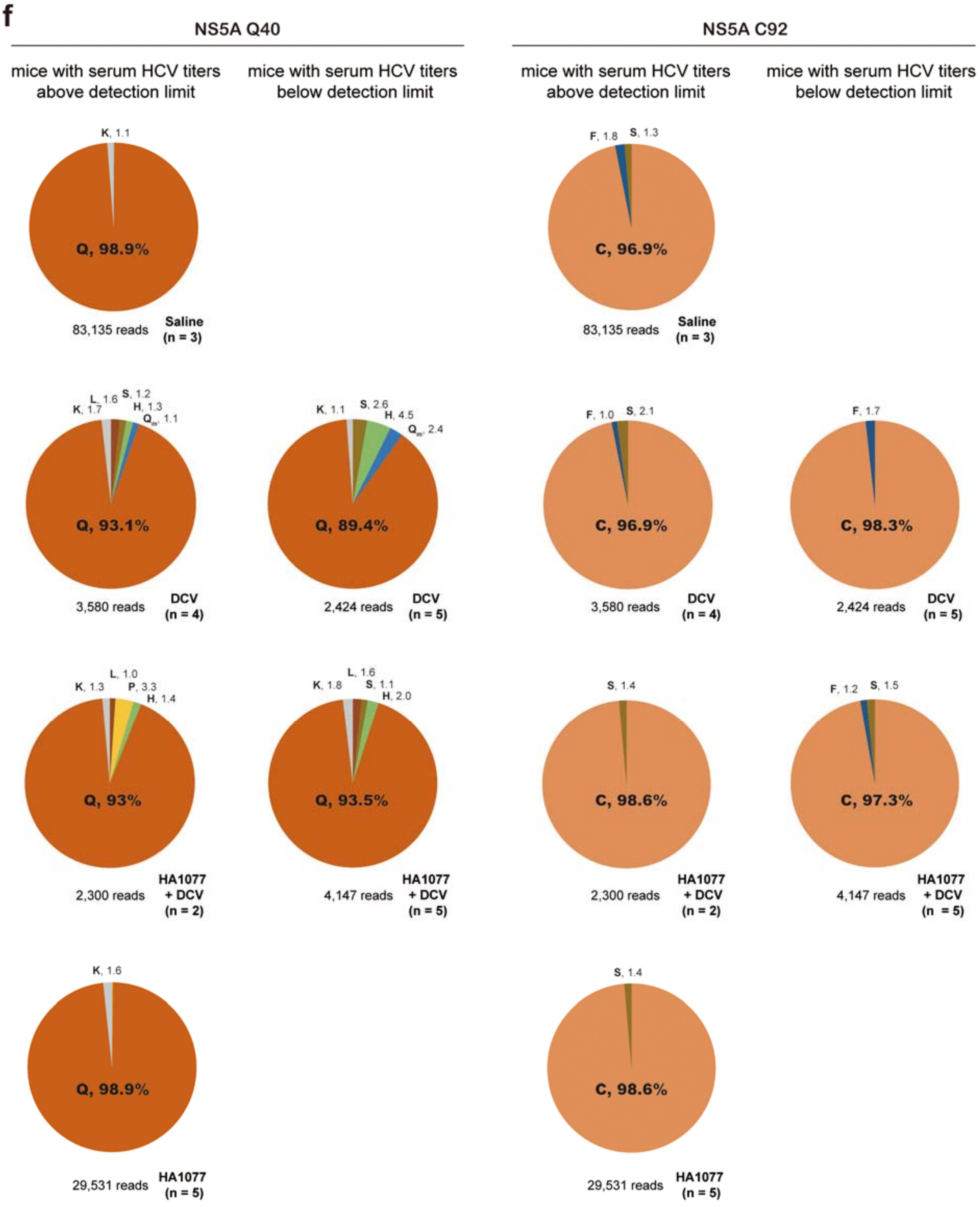

NS5A Q40
NS5A C92
mice with serum HCV titers above detection limit
mice with serum HCV titers below detection limit
mice with serum HCV titers above detection limit
mice with serum HCV titers below detection limit
K, 1.1
Q, 98.9%
83,135 reads
Saline (n = 3)
F, 1.8
S, 1.3
C, 96.9%
83,135 reads
Saline (n = 3)
K, 1.7
L, 1.6
S, 1.2
H, 1.3
Qm, 1.1
Q, 93.1%
3,580 reads
DCV (n = 4)
K, 1.1
S, 2.6
H, 4.5
Qm, 2.4
Q, 89.4%
2,424 reads
DCV (n = 5)
F, 1.0
S, 2.1
C, 96.9%
3,580 reads
DCV (n = 4)
F, 1.7
C, 98.3%
2,424 reads
DCV (n = 5)
K, 1.3
L, 1.0
P, 3.3
H, 1.4
Q, 93%
2,300 reads
HA1077 + DCV (n = 2)
K, 1.8
L, 1.6
S, 1.1
H, 2.0
Q, 93.5%
4,147 reads
HA1077 + DCV (n = 5)
S, 1.4
C, 98.6%
2,300 reads
HA1077 + DCV (n = 2)
F, 1.2
S, 1.5
C, 97.3%
4,147 reads
HA1077 + DCV (n = 5)
K, 1.6
Q, 98.9%
29,531 reads
HA1077 (n = 5)
S, 1.4
C, 98.6%
29,531 reads
HA1077 (n = 5)

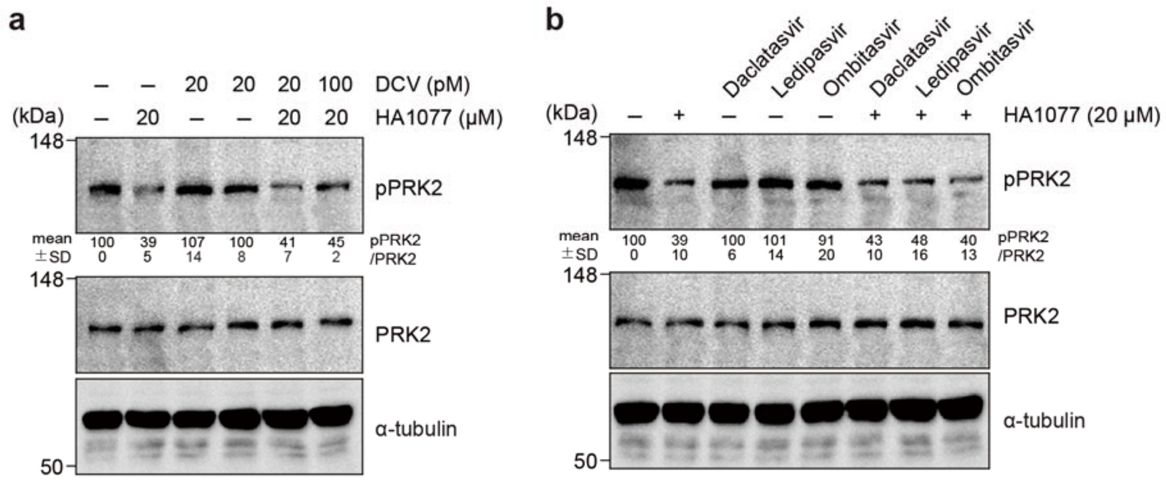

Supplementary Figure 6. Impact of NS5A inhibitors on PRK2 activation. (**a**) The Huh7-derived R-1 cell line harboring an HCV GT1b subgenomic replicon was treated with the indicated concentrations of DCV and/or HA1077 for 30 min. (**b**) R-1 cells were treated with the indicated NS5A inhibitors (20 pM) with or without HA1077 (20 μM) for 2 h. Total cell lysates were analyzed by immunoblotting for the indicated proteins. Phosphorylated form PRK2 was detected using rabbit polyclonal antibodies against pPRK2(Thr816). The immunoblotting images shown in (**a**,**b**) are from one representative experiment of three independent experiments with similar results. Relative signals (% of pPRK2/PRK2 level in mock-treated cells) determined as in Supplementary Fig. 1 are shown below a representative blot.

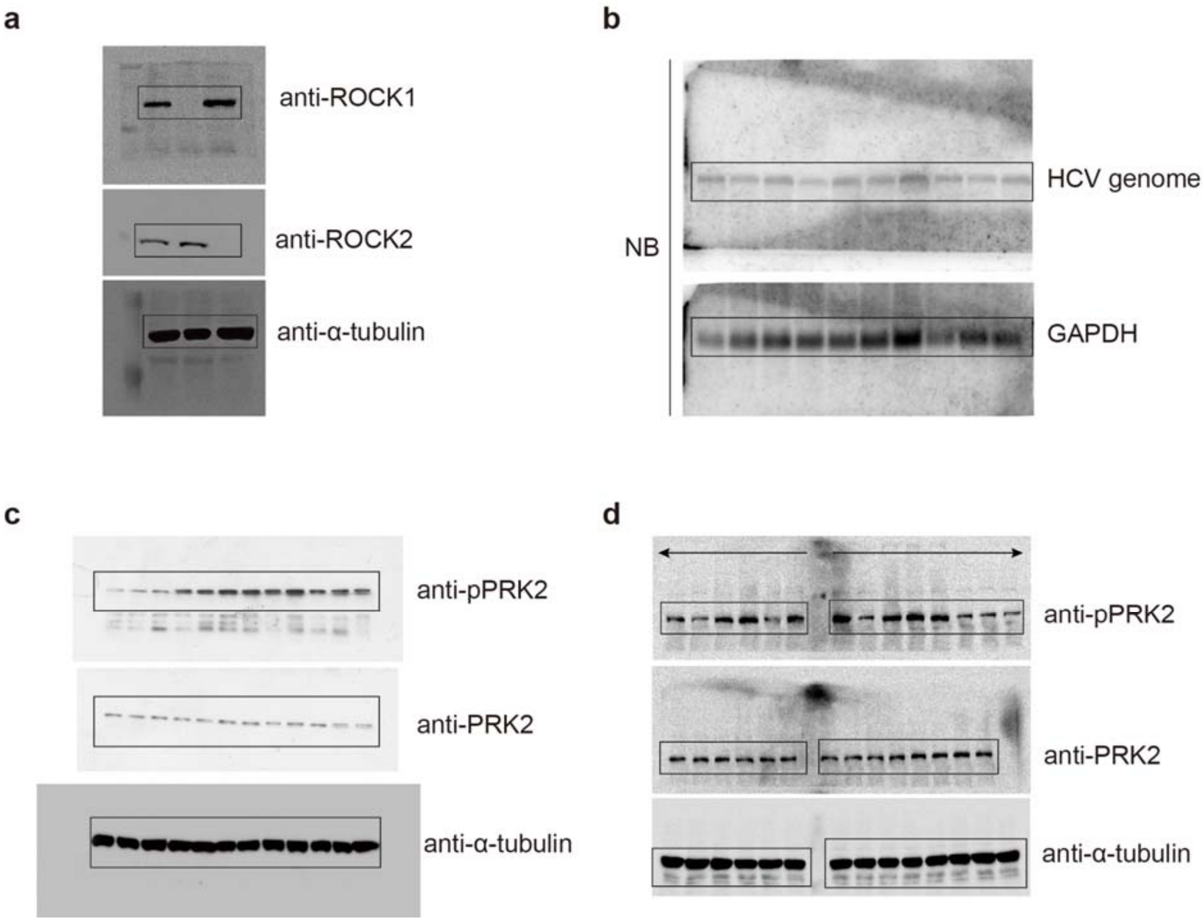

Supplementary Figure 7. Uncropped images of immunoblots and northern blots. (**a**) For Fig. 1f. (**b**) For Fig. 7c. (**c**) For Supplementary Fig. 1. (**d**) For Supplementary Fig. 6.

Supplementary Table

Supplementary Table 1. Primers used in this study

Primer*	5′–3′
F28S F	CTG ACC TCT AAA TTG TCC CCC AAG CTG CCC GGC
F28S R	GCC GGG CAG CTT GGG GGA CAA TTT AGA GGT CAG
L31D F	CTA AAT TGT TCC CCA AGG ACC CCG GCC TCC CCT TC
L31D R	GAA GGG GAG GCC GGG GTC CTT GGG GAA CAA TTT AG
L31H F	CTA AAT TGT TCC CCA AGC ACC CCG GCC TCC CCT TC
L31H R	GAA GGG GAG GCC GGG GTG CTT GGG GAA CAA TTT AG
L31M F	CTA AAT TGT TCC CCA AGA TGC CCG GCC TCC CCT TC
L31M R	GAA GGG GAG GCC GGG CAT CTT GGG GAA CAA TTT AG
L31Q F	CTA AAT TGT TCC CCA AGC AGC CCG GCC TCC CCT TC
L31Q R	GAA GGG GAG GCC GGG CTG CTT GGG GAA CAA TTT AG
L31S F	CTA AAT TGT TCC CCA AGT CGC CCG GCC TCC CCT TC
L31S R	GAA GGG GAG GCC GGG CGA CTT GGG GAA CAA TTT AG
L31V F	CTA AAT TGT TCC CCA AGG TGC CCG GCC TCC CCT TC
L31V R	GAA GGG GAG GCC GGG CAC CTT GGG GAA CAA TTT AG
C92R F	GAC CTT TCC TAT CAA TCG CTA CAC GGA GGG CCA G
C92R R	CTG GCC CTC CGT GTA GCG ATT GAT AGG AAA GGT C
Y93H F	CTT TCC TAT CAA TTG CCA CAC GGA GGG CCA GTG
Y93H R	CAC TGG CCC TCC GTG TGG CAA TTG ATA GGA AAG

* F, forward primer; R, reverse primer
